# Contaminant Back Diffusion from Low-Conductivity Matrices: Case Studies of Remedial Strategies

**DOI:** 10.3390/w15030570

**Published:** 2023-02-01

**Authors:** Julie Blue, Thomas Boving, Mary Ellen Tuccillo, Jonathan Koplos, Jason Rose, Michael Brooks, David Burden

**Affiliations:** 1 Eastern Research Group, Concord, MA 01742, USA; 2 Department Geosciences/Department Civil and Environmental Engineering, University of Rhode Island, Kingston, RI 02881, USA; 3 PG Environmental, Golden, CO 80401, USA; 4 U.S. Environmental Protection Agency, Ada, OK 74820, USA

**Keywords:** back diffusion, remediation, CVOCs, rebound, low-conductivity zone, fractured rock, aquitard

## Abstract

Recalcitrant groundwater contamination is a common problem at hazardous waste sites worldwide. Groundwater contamination persists despite decades of remediation efforts at many sites because contaminants sorbed or dissolved within low-conductivity zones can back diffuse into high-conductivity zones, and therefore act as a continuing source of contamination to flowing groundwater. A review of the available literature on remediation of plume persistence due to back diffusion was conducted, and four sites were selected as case studies. Remediation at the sites included pump and treat, enhanced bioremediation, and thermal treatment. Our review highlights that a relatively small number of sites have been studied in sufficient detail to fully evaluate remediation of back diffusion; however, three general conclusions can be made based on the review. First, it is difficult to assess the significance of back diffusion without sufficient data to distinguish between multiple factors contributing to contaminant rebound and plume persistence. Second, high-resolution vertical samples are decidedly valuable for back diffusion assessment but are generally lacking in post-treatment assessments. Third, complete contaminant mass removal from back diffusion sources may not always be possible. Partial contaminant mass removal may nonetheless have potential benefits, similar to partial mass removal from primary DNAPL source zones.

## 1. Introduction

Diffusion has been recognized since the mid-1970s as an important process controlling contaminant transport in low-conductivity zones (LCZs), such as unconsolidated clay-rich deposits and aquitards or the matrix of fractured bedrock aquifers [1–7]. Recent reviews summarize the current state of knowledge on this issue as reflected in the relevant literature [8,9]. Diffusive transport is driven by concentration gradients, which orient diffusive flux from high to low concentrations. The hydraulic conductivity distribution can also provide useful context for understanding diffusive flux. Diffusive flux from high-conductivity zones (HCZs; e.g., sand and gravel deposits or bedrock fractures) to LCZs is called forward diffusion and results in the accumulation of contaminants in the LCZs. This process occurs relatively early in the lifecycle of a contaminated site as contaminant mass from the primary source (e.g., dense non-aqueous phase liquid (DNAPL)) is transported by advection and dispersion through the HCZs. Mass transfer through forward diffusion is proportional to the residence time of higher-concentration contaminants in the HCZ (e.g., [10]). However, as the original source strength decreases, either through natural attenuation or remedial intervention, the concentration reductions in the HCZ lead to a reversal of the concentration gradient, resulting in back diffusion of the contaminant from the LCZ to the HCZ (e.g., [11,12]).

Plume persistence at contaminated groundwater sites often refers to the continued presence of contaminants at concentrations exceeding a remedial goal. It generally signifies a source of contaminant mass that has eluded initial characterization or remedial efforts. Plume persistence may also reflect rate-limited processes associated with contaminant mass removal and limitations in naturally occurring abiotic/biotic degradation mechanisms. Contaminant rebound typically refers to an increase in contaminant concentrations immediately following remedial treatment induced reductions in contaminant concentration. Rebound results from remaining contaminant, despite remedial treatment, and one or more processes acting to delay equilibrium conditions at the sampling device, such as rate-limited desorption, rate-limited DNAPL dissolution (e.g., [13]), or contaminant travel time through a clean region created by treatment to reach the sampling device (which may be exacerbated by slow advection or diffusion). While back diffusion may not be the sole reason for contaminant rebound or plume persistence, numerous laboratory and modeling studies have demonstrated that back diffusion can be, if not the sole factor, then a significant factor for both [7,14–17]. Moreover, several field studies have demonstrated plume persistence due to back diffusion after primary source zone isolation [14,18].

Proper characterization of site hydrogeology and contaminant distribution provides crucial information to evaluate the significance of back diffusion. Soil cores have traditionally provided rigorous characterization data and continue to do so (e.g., [11,18,19]). However, vertical spacing is important. For instance, Parker et al. [18] determined that a thin clay layer < 0.2 m thick, easily overlooked during coring with large vertical sample spacing, provides enough storage capacity for dissolved and sorbed contaminants to create several decades of plume persistence. Fractured media may be particularly susceptible to issues arising from back diffusion, and characterization of fractured media sites has been discussed by Parker et al. [20]. More recently, a variety of field tests specific to back diffusion have been proposed [19,21–24]. In addition, new approaches to numerical modeling have been developed to better simulate back diffusion [25–29].

Forward diffusion results in the accumulation of contaminants in LCZs where they cannot be easily reached by remedial strategies that depend exclusively on groundwater flow for flushing contaminants or delivering reactants (e.g., [16,30]). Conventional pump and treat (PAT) systems have historically been the most common remedial option for which a long period of record exists at many sites. Data from long-term monitoring of these sites often demonstrate the effects of plume persistence due to back diffusion (e.g., [31,32]). This often significantly influences the time needed to achieve remedial goals such as maximum contaminant levels (MCLs). In some cases, it may take decades or longer (e.g., [18,30]). In this context, Hou and Al-Tabbaa [33] noted that secondary environmental effects, such as increases in greenhouse gas emissions resulting from long-term remedial activities, are significant.

Amendment injection remediation technologies commonly used in HCZs, such as in situ chemical oxidation (ISCO), have had limited success (e.g., [34]) because reactive reagents fail to penetrate sufficiently deep into LCZs or are quickly flushed out of the treatment zone (e.g., [15]). Cavanaugh et al. [16] suggest that complete destruction of LCZ contaminants may be impracticable and note that alternative strategies include partial LCZ treatment and active treatment at the HCZ/LCZ interface. On the other hand, Baker et al. [35] summarized thermal treatment of 10 different source zones across five sites and highlighted that back diffusion may not always limit the benefits obtained from nonaqueous phase liquid (NAPL) source treatment. Moreover, several studies have noted the importance of LCZ abiotic and biotic degradation processes (e.g., [36–40]). Wanner et al. [41] point out the importance of compound-specific isotope analysis (CSIA) as a diagnostic tool to demonstrate LCZ degradation.

Horst et al. [42] and Brooks et al. [9] have presented reviews of remedial technologies and strategies aimed at addressing contamination in LCZs and plume persistence due to back diffusion. The latter divided back diffusion remediation technologies into four major categories, namely (1) passive LCZ management approaches, such as PAT, managed natural attenuation, or permeable reactive barriers; (2) approaches that involve movement of amendments into the LCZ to break down contaminants before they can back diffuse to the HCZ; (3) approaches that change the physical characteristics of the secondary source, such as soil fracturing and mixing, or viscosity modifications; and (4) thermal and electrokinetic remediation technologies. In general, the success and cost of these remediation technologies are currently difficult to evaluate because, as noted by Brooks et al. [9], there is a lack of reports on field-based studies that specifically address back diffusion treatment.

The focus herein is on pilot- and field-scale demonstrations of LCZ remediation schemes to mitigate back diffusion and plume persistence. Explored are remedial approaches, levels of success, and lessons learned. Technology costs and general applicability considerations were not considered. The description of our approach for identifying peer reviewed literature and other sources is followed by a review of four salient case studies. Improving remedial efficiency for sites with back diffusion promises to save costs, decrease greenhouse gas emissions associated with remedial activities, and—most important—provides greater certainty that groundwater resources are not imperiled by persistent contamination. Therefore, this work should be of interest to remedial site managers, regulators, and remedial technology developers.

## 2. Methodology

A literature search was conducted using Google, Google Scholar, Web of Science, Science Direct, and Wiley. The searches were restricted to the English language and used variations and combinations of three key search terms to identify potential case studies: groundwater, remediation, and back diffusion. A focus was placed on identifying peer-reviewed literature and technical reports from government programs (United States Geological Survey (USGS), The Department of Defense’s Strategic Environmental Research and Development Program (SERDP) and the Environmental Security Technology Certification Program (ESTCP), etc.). Gray literature, such as presentations, conference abstracts, and non-peer-reviewed papers and reports by state and federal agencies, were also considered. Two main criteria were used to prioritize search results for the selection of case studies: (1) sites with characterization data to support the existence of plume persistence due to back diffusion and (2) sites where one or more remedial strategies were applied to address back diffusion, at either pilot or field scales. Interviews with site-responsible parties were also conducted in some cases to collect more information.

## 3. Case Studies

The literature review showed that back diffusion appears to be a factor impeding remediation at many sites. Moreover, varying remedial actions have been taken to address the problem. However, the level of information available describing site contamination, evidence for the importance of and initiation of back diffusion, remedial actions taken to address it (by targeting contamination in LCZs), and the performance of the remedial technologies used at most of these sites was too limited for extensive discussion. Consequently, many sites were eliminated as case studies because they lacked data to specifically identify back diffusion or focus on back diffusion in the context of a remedial technology. Another challenge in selecting case studies was that some sites lacked identification (i.e., anonymous sites), which prevented evaluation of current conditions.

Table 1 provides a list of sites that were selected for additional review after an initial screening but were ultimately not selected for in-depth case studies. Table 2 provides a summary of the four case studies selected to demonstrate important issues related to back diffusion and remedial options for addressing it. A high level of technical information was available for these sites in the literature. Both tables summarize site geology, remediation technologies, and key points learned. The sites listed in Table 1 generally had comparatively less field-based information on site remediation or were deemed to have considerable overlap with remedial technologies used at the sites selected for case studies. Overall, the case studies summarized in Table 2 and discussed in detail below cover a range of remediation approaches and geological settings.

### 3.1. Case Study: Cocoa, FL

Located in Cocoa, Florida, the Precision Fabricating & Cleaning (PFC) site is a small metal fabricating and cleaning facility with a history of trichloroethylene (TCE) DNAPL releases. This site has been the subject of several studies, including one on the nature of DNAPL contaminants in the subsurface [57], mass flux distributions from DNAPL source zones [59], and back diffusion from thin LCZs [18,60].

#### 3.1.1. Site Geology and Hydrogeology

The site geology results from a complex sequence of coastal deposition processes. The uppermost 8.5 to 9.1 m below ground surface (bgs) is composed of homogenous, well-sorted beach sand with scattered lenses of coquina (limestone composed of cemented shell fragments). In the source area, upper and lower clay layers are found at 9.1 and 10.7 m bgs, respectively, separated by a sandy layer. Towards the southwest end of the site, the upper and lower clay beds seem to converge [80]. However, Parker et al. [18] note that one clay layer extends continuously across the source area and in the downgradient direction of the plume, ranging in depth from 8 to 10 m with a thickness of 5 to 20 cm.

**Figure 1.**
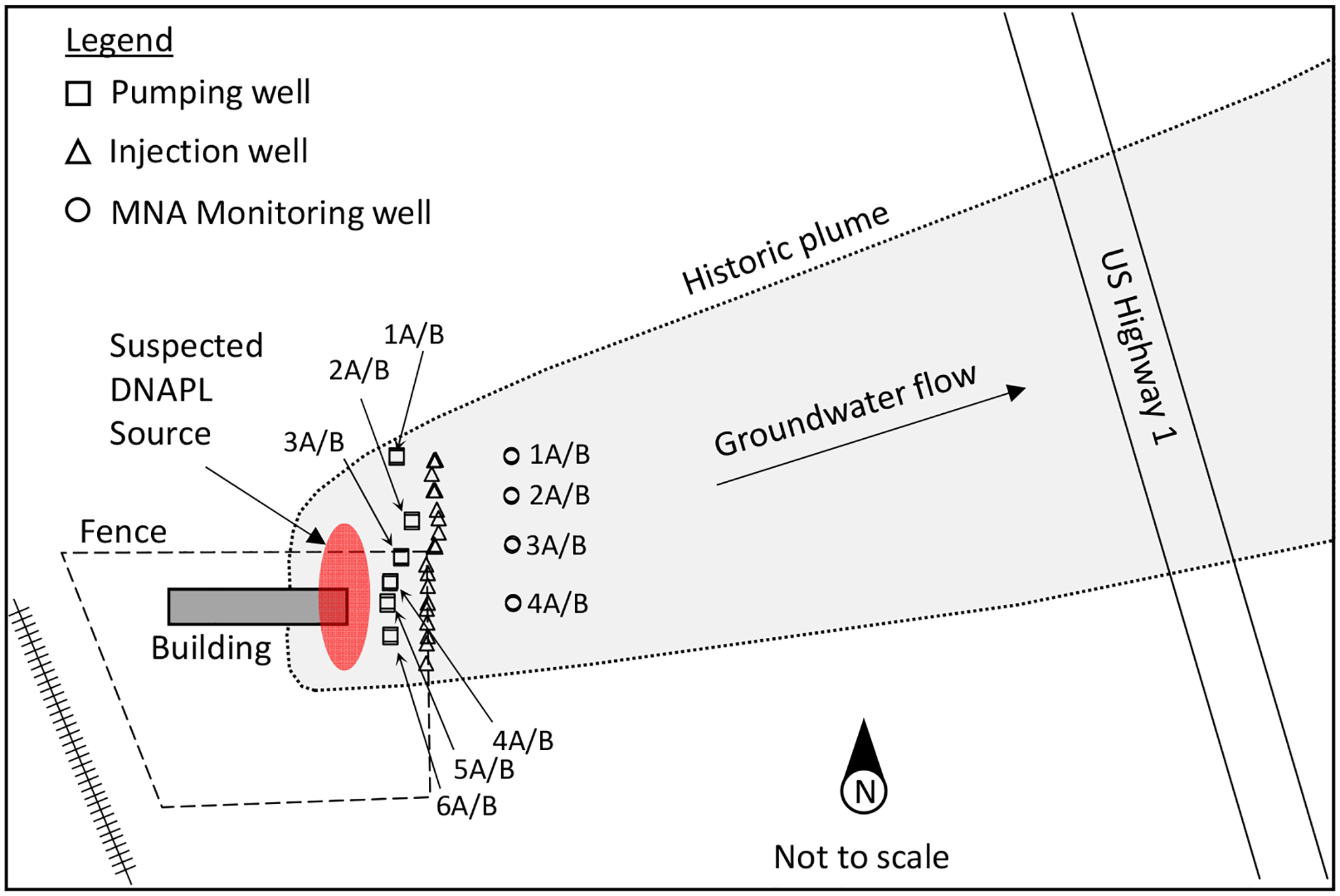
Conceptual PFC site map at Cocoa, Florida, showing source area in red, and contaminant plume in gray. (Adapted from Parker et al. [18] and Geosyntec [62]).

Directly under the deeper clay layer, there is a softer, shell-rich clay unit that coarsens with depth, extending to about 12 m bgs. From about 12 to 15 m bgs, there is an organic-rich unit consisting of poorly sorted brown sands, silts, and sandy, shell-rich layers. Interbedded with the sandy, shell-rich beds are poorly sorted sand and silt layers [80]. Depth to groundwater is approximately 3 m. The groundwater flow direction is generally to the east–northeast and the contaminant plume likewise extends in this direction (Figure 1).

#### 3.1.2. Nature of Contamination

Multiple spill events occurred between 1964 and 1977 while TCE was used at the site. In addition to smaller, routine spills, two larger spills contributed to the contamination of the underlying aquifer. In 1966, two 55-gallon drums spilled, and in 1977, a hose burst [59]. A DNAPL source below the facility building (Figure 1) created a plume of TCE and its degradation products (cis-1,2-dichloroethene [cDCE], trans-1,2-dichloroethene, and vinyl chloride [VC]) which extended ~800 m downgradient. As of November 2021, cDCE and VC were the dominant contaminants above target cleanup levels across most of the site, while TCE exceeded target cleanup levels in only a few locations.

#### 3.1.3. Remedial History

The treatment system selected for the site consisted of hydraulic isolation of the DNAPL source zone with monitored natural attenuation in the downgradient plume. Source-zone hydraulic isolation was achieved using a PAT system composed of 12 extraction wells located along a north–south transect immediately downgradient of the primary source zone. Half of the extraction wells were screened above the uppermost clay layer (above ~9 m bgs), or in the shallow zone, and the remaining extraction wells were screened below the uppermost clay layer, or in the deep zone. Water extracted from these wells was treated and injected into nineteen injection wells (twelve in the shallow zone, seven in the deep zone) located along another north–south transect ~20 m downgradient from the pumping well transect. The intent of this design was to hydraulically isolate the primary source zone, facilitate plume detachment with a wedge of clean water, and enhance flushing of the downgradient plume.

The PAT system operated from August 2002, extracting 207 m^3^/day, until November 2006, when adjustments were made to optimize the operation, including termination of wells that were consistently below MCLs and increasing extraction rates in areas with higher volatile organic compound (VOC) concentrations. The total modified pumping rate after these adjustments was 163 m^3^/day. Groundwater VOC concentrations downgradient of the source area initially declined but remained above remedial goals in most cases. In 2008, the potential causes of plume persistence observed at the site were investigated [18]. Soil core samples were collected from four locations on the property at closely spaced depth intervals of 2.5 to 5 cm and analyzed for VOCs. The results showed that VOC concentrations within the clay layers exceeded concentrations in the surrounding sandy layers by ~20 to ~300 μg/g, and that these changes occurred over small distances (~10′s of cm). These features are consistent with an explanation of plume persistence due to back diffusion.

**Figure 2.**
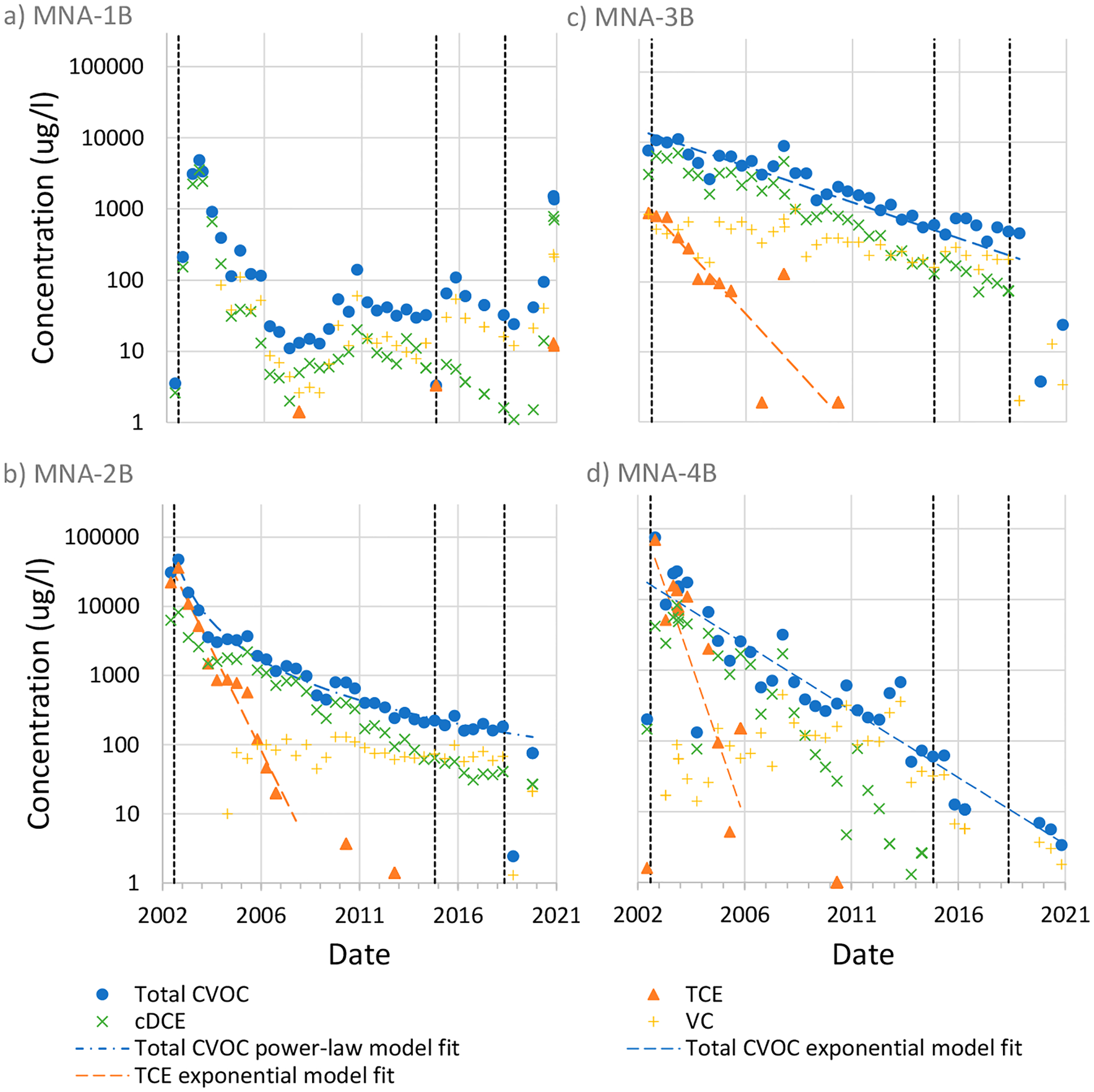
Concentration–time series for monitoring wells (**a**) MNA-1B, (**b**) MNA-2B, (**c**) MNA-3B, and (**d**) MNA-4B at the PFC site in Cocoa, Florida. Model fits are shown for select data only. The power law model fit for total CVOC in panel (**b**) is based on an exponent of 2. The vertical dashed lines indicate the following events from left to right: start of the PAT system in 2002, first biotreatment injection in 2015, and second biotreatment injection in 2019. Data shown in the graphs were taken from Geosyntec [63].

Another component of the evaluation completed by Parker et al. [18] was analysis of concentration-time series in downgradient monitoring wells. Figure 2 shows concentration–time series data from four monitoring wells included in that analysis and provides an update to the information presented in Parker et al. [18]. The wells shown in Figure 2 are screened in the deep zone and are aligned in a north–south transect roughly 40 m downgradient of the injection well transect. Advective travel time estimates from the injection well transect to the monitoring well transect range from <0.5 to <1.5 years [18], yet concentration tailing is evident despite exceeding travel time estimates by more than a decade. However, the tailing is predominantly evident in the daughter products cDCE and VC. The parent TCE compound has a distinctly different pattern with less persistence, at least in three of the wells shown. One explanation for this is that the hydraulic containment system was effective in capturing source TCE, while the longer residence time associated with diffusion and/or geochemical conditions in the clay may permit degradation of TCE to occur before reaching the monitoring well transect. Parker et al. [18] note that the clayey LCZs at the site may be more conducive to reductive dechlorination than the sandy HCZs.

**Figure 3.**
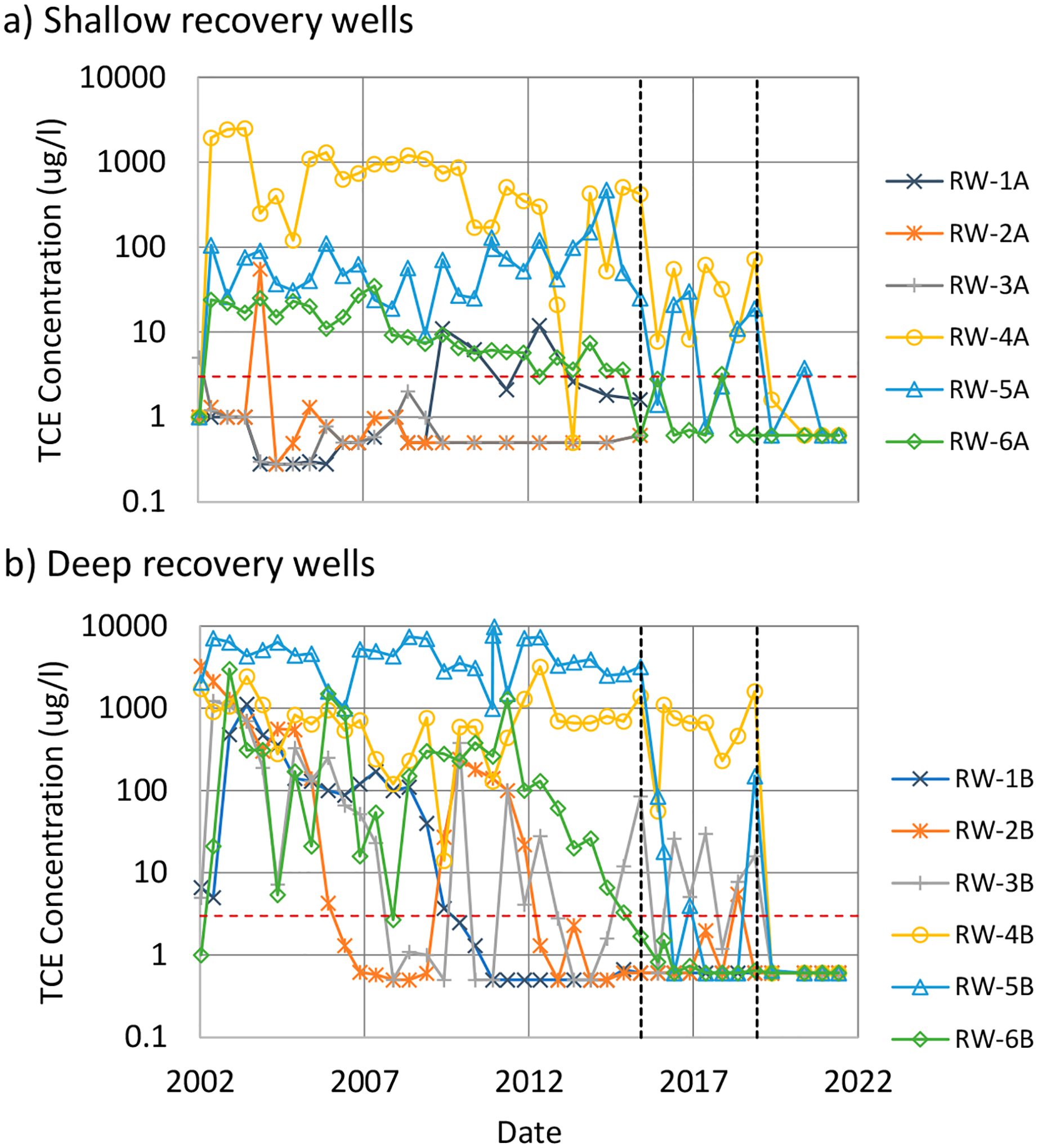
Concentration–time series for TCE from (**a**) shallow and (**b**) deep recovery wells used to hydraulically isolate the primary source zone. The vertical dashed lines indicate the first biotreatment injection in 2015 and second biotreatment injection in 2019. The red horizontal dashed line indicates the TCE remediation goal. Data shown in the graphs were taken from Geosyntec [63].

In January 2015, groundwater concentrations in water samples collected from bundled wells in the source area (upgradient of the recovery well transect) had changed significantly since they were last sampled in 2005. For example, TCE concentrations in one bundle well increased from 5.3 μg/L in 2005 to 200,000 μg/L in 2015. Consequently, a portion of the source zone was treated by biostimulation/bioaugmentation in October 2015. An emulsified soya bean oil, which acts as an electron donor to promote anaerobic biodegradation, was injected together with a *Dehalococcoides* microbial culture to promote reductive dechlorination. These amendments were injected into the source area at depth intervals of 9.1 to 10.4 m, 10.7 to 11.9 m, and 12.5 to 13.7 m [61]. Significant reductions in concentration were evident in the recovery well effluent following these injections (Figure 3). Contaminant mass recovery from the PAT system decreased to the point that site-responsible parties temporarily discontinued the PAT system in May 2019. That same month, a second series of injections were completed at multiple locations in the source zone and at locations in the downgradient plume.

The combined result of these injections was a significant reduction in source zone concentrations by up to four orders of magnitude (e.g., Figure 3). Responsible parties at the site are currently evaluating periodic biostimulation/augmentation injections as a replacement to the PAT system. It remains to be seen, however, to what extent these activities will mitigate plume persistence due to back diffusion in the plume. While there is evidence of concentration reductions at MNA-2B and MNA-3B in Figure 2 (for example), more monitoring data are needed to better assess the longevity of these reductions.

#### 3.1.4. Lessons Learned

The PFC site illustrates challenges often encountered when remediating DNAPL sites consisting of interbedded LCZ clays and HCZ sands. Operation of the PAT system to isolate the DNAPL source did not eliminate the contaminant plume in the time frame initially expected. Nonetheless, over the 17 years it was in operation, it did prevent ~730 kg (~500 L) of equivalent TCE [63] from entering the downgradient plume. Moreover, the data from downgradient monitoring wells suggest that it was effective in isolating the source as intended.

Concentration–time series data from select locations downgradient of the extraction/injection transects indicate different patterns for the parent TCE compared to the daughter products cDCE and VC. The parent TCE shows a larger rate of decrease compared to cDCE and VC. One explanation for this is that the DNAPL source is predominantly TCE, and the more rapid decline in TCE compared to cDCE and VC reflects hydraulic isolation of the source due to the PAT system, and degradation downgradient of the extraction/injection transects. It is uncertain if degradation occurs in the HCZ, LCZ, or both, but Parker et al. [18] suggest that the clayey LCZs are more conducive to degradation than the HCZs. Assuming that to be the case, then TCE discharged from the source zone prior to hydraulic containment would have diffused into the LCZ, undergone degradation, and then the diffusion of cDCE and VC from the LCZ would result in the observed tailing. Moreover, the rates of cDCE and VC degradation are apparently much less than TCE, and relatively longer than the time scale for mass flux from the LCZ.

Replacement of the PAT for hydraulic contaminant with periodic amendment injection for biostimulation/bioaugmentation is currently being evaluated at the site. Results in the source zone indicate significant reductions in concentration after initial injections. Assuming a LCZ dominated by diffusive transport, it should be recognized that amendment contact with contaminant in the LCZ will also be limited by diffusive transport. Nonetheless, the processes that act to promote contaminant mass transfer from the HCZs to the LCZs at early times (i.e., large concentration gradients directed into the contaminant-free LCZ) should also promote amendment mass transfer into the initially amendment-free LCZs. Moreover, the timeframe for diffusional transport through the entire thickness of the LCZ will be less for thin layers compared to thicker layers.

### 3.2. Case Study: Jacksonville Naval Air Station

The Jacksonville Naval Air Station (NAS), part of the larger Jacksonville Naval Complex, is located approximately 13 km south of Jacksonville, Florida. An environmental investigation of the Jacksonville NAS started in 1979 and identified potential sources of contamination, including the base’s former dry cleaner, which operated from 1962 to 1990. This location has been the subject of several environmental research studies, including one on alternative endpoints at challenging sites [81], natural attenuation of NAPL source zones [82], high-resolution soil core sampling for generating source history [19,52], and electrokinetic (EK)-enhanced bioremediation [64,65]. The works reported by Adamson et al. [19], Cox et al. [64], and Meinel et al. [65] are the primary basis for this case study.

#### 3.2.1. Site Geology and Hydrogeology

A veneer of surface fill covers interbedded layers of sand, clayey sand, sandy clay, and clay down to about 46 m bgs. An upper surficial sandy aquifer is underlain by a clay unit at depths of 3.0 to 6.1 m bgs, with a transition zone of clayey sand or sandy clay between them in the area where the former dry cleaner was located [19]. The clay layer provides lower confinement for the surficial sands and is the unit into which the CVOCs have diffused. The water table is between approximately 1 and 2 m bgs, and groundwater in the surficial aquifer flows to the east. The average hydraulic conductivity in the shallow sand near the pilot site was estimated to be 5 × 10^−3^ cm/s [82].

#### 3.2.2. Nature of Contamination

The former dry cleaning facility released PCE through leaks and spills that contaminated the shallow aquifer, and DNAPL has been identified in the subsurface [83]. Aqueous phase PCE has been detected in the sandy aquifer along with its degradation products (primarily TCE, cDCE, and VC). Historic concentrations in the surficial aquifer for PCE, TCE, and cDCE ranged from approximately 1000 μg/L to 10,000 μg/L [83], while concentration for VC ranged from ~100 μg/L to >2000 μg/L [84].

As part of the study to estimate the contamination release history from CVOC distributions in the LCZ, Adamson et al. [19] collected soil core samples at high-resolution vertical intervals (at least every 30 cm, and 5 to 15 cm within LCZs and the HCZ/LCZ interface) using direct push techniques. Soil cores were taken from four locations ranging from ~6 m to ~130 m downgradient of the source area. Samples were analyzed for CVOCs, porosity, organic carbon, and genetic biomarkers of *Dehalococcoides* (*Dhc*) and *vinyl chloride reductase* (*vcrA*).

In general, the highest soil CVOC concentrations were detected between 4 and 6 m bgs at the interface between the mostly sandy sediments above and the clay below. Moreover, 80% of the total CVOC mass was in the LCZ clay and in the overlying transition zone. The results also showed that PCE is the main soil contaminant in most areas, except for the location furthest downgradient, where cDCE was the dominant CVOC. No evidence was found for significant biodegradation in the LCZ.

The PCE peak soil concentration was ~30 μg/g and occurred in the core location closest to the source zone. Because the peak was in the LCZ but close to the interface, and because the PCE spatial distribution in the LCZ suggested diffusional transport, it is likely that back diffusion was just beginning to occur at the time of sampling. Adamson et al. [19] note that the peak CVOC concentration at the furthest downgradient location was cDCE at 4.5 μg/g, and that it occurred in the HCZ sandy unit. Consequently, this location was still under the action of forward diffusion at the time samples were collected. The predominance of cDCE at this location suggested that degradation occurs primarily in the downgradient HCZ and is perhaps limited due to high contaminant levels closer to the source area.

#### 3.2.3. Remedial History

Air sparging and soil vapor extraction (AS/SVE) in the permeable surficial aquifer were initially chosen as the remedial strategy for this part of Jacksonville NAS. The AS/SVE system was shut down around 2005 because it was no longer considered effective [84]. Subsequent work included a 2013 pilot test for in situ bioremediation in the HPZ [85], and a demonstration from 2014 to 2017 to evaluate an innovative EK process to distribute bioaugmentation and biostimulation amendments through the LCZ clay layer [64,83,86]. The EK treatment area was located within and is a small (12 m by 12 m) portion of the in situ bioremediation treatment area completed in 2013. The two projects were conceived and conducted independent of each other. The goal of treating the site with EK-enhanced amendment delivery, which is the focus of this case study, was to promote the distribution of the amendments within the formation due to electrical gradients, overcoming the transport limitations of LCZ materials. This in turn would facilitate in situ biodegradation of CVOCs that had diffused into the clayey LCZ, thereby preventing their release back into the sandy HCZ via back diffusion [64].

**Figure 4.**
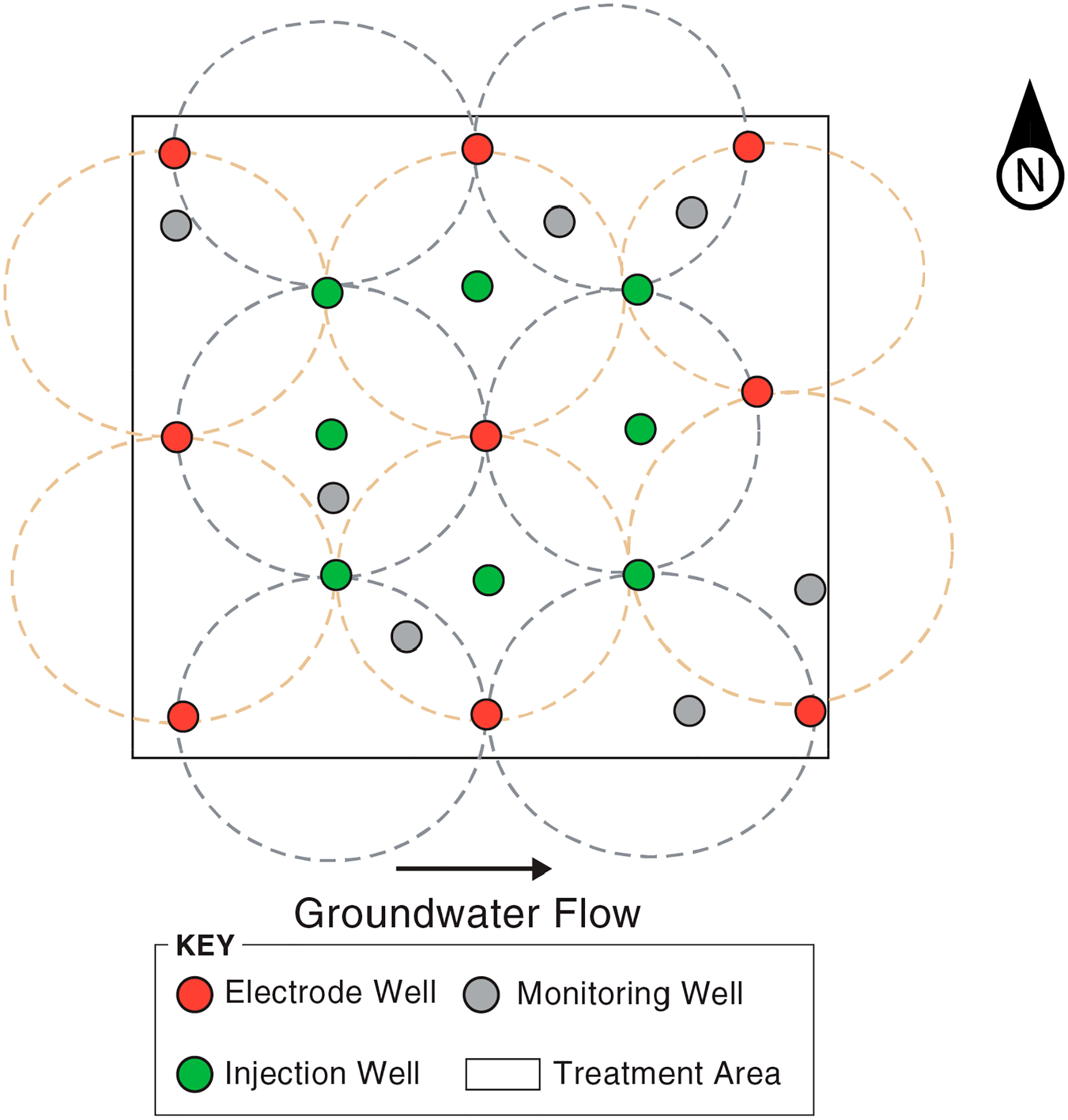
Well layout in the 12 m by 12 m EK treatment area with electrodes (red circles), amendment injection well (green circles), and monitoring wells (gray circles). The electric fields during Stages 1 and 2 are conceptually represented by the blue dashed lines and orange dashed lines, respectively. Adapted from Cox et al. [64].

The electrodes, amendment injections wells, and monitoring wells associated with the EK system are shown in Figure 4. An additional four monitoring wells were located outside the treatment area (not shown). All wells were screened within the clay from 5.8 to 7.0 m bgs. After well installation, baseline groundwater characterization of the treatment zone was conducted in October 2014. Samples were analyzed for a suite of parameters, including metals, major anions, CVOCs, total organic carbon (TOC), volatile fatty acids (VFAs), and dissolved hydrocarbon gases. Carbon-based constituents (TOC and VFAs) allow for monitoring the distribution of electron donors. Microbiological parameters *Dhc*, *Dehalobacter* (*Dhb*), and *vcrA* were also measured as indicators of the potential for reductive dehalogenation of CVOCs. The baseline data showed low TOC and VFAs, and virtually no *Dhc*, *Dhb*, or *vcrA*.

Baseline soil samples were collected at depths of 5.6 m, 6.4 m, and 7.0 m bgs. The sample at 5.6 m is near the top of the clay interface, while the samples at 6.4 m and 7.0 m are within the clay. Samples were analyzed for metals, CVOCs, *Dhc*, *Dhb*, and *vcrA*, as well as grain size analysis. As with the groundwater samples, the soil analyses showed no evidence of reductive dechlorination. Other tests included soil mineralogy and its zeta potential.

As noted elsewhere (e.g., [87,88]), the three mechanisms that can promote the migration of amendments during EK treatment are (1) electromigration (ionic species move through water under the influence of an electric field), (2) electroosmosis (the pore fluid itself moves due to the movement of ions in the double layer), and (3) electrophoresis (charged particles and bacteria move through water under the influence of an electric field). The relative importance of these processes depends on the contaminants and the subsurface characteristics; the surface charge of clay materials also contributes to the effectiveness of electroosmosis in clays and silts [89]. A treatability study was carried out that included an evaluation of the potentials for both electromigration and electroosmosis [64]. The rate of electromigration was estimated at 3.3 cm/day or higher based on bench-scale column tests with bromide. For electroosmosis, the rate of flow is related to the zeta potential and is affected by the ionic strength and pH of the water. Measurements of the zeta potential in clay materials from the treatment area indicated that the pH in the electrode wells should be maintained above 5 for adequate efficiency, and the EK system allows for pH control.

The EK demonstration began with a 60-day period for pH conditioning of the treatment zone via sodium carbonate solution addition. Subsequently, there were two operational stages with six months of incubation between them. The stages used different anode and cathode configurations to orient the electrical fields 90 degrees from each other and thereby promote amendment distribution. In Stage 1, lactate, an electron donor to support microbial reductive dehalogenation, was supplied to the electrode wells and amendment supply wells in short pulses several times per day. Also injected were pH buffer, acid, and base additions as needed to maintain pH in a favorable range. Following approximately 75 days of operation, the system was shut down for 48 h for KB-1 bioaugmentation. The KB-1 microbial culture that contains *Dhc* was added to both electrode and amendment injection wells to provide a microbial consortium capable of reductive dehalogenation of COVCs. Following bioaugmentation, EK operation resumed and continued for about five months. Monitoring samples were then collected, followed by a 6-month incubation period. Similarly to Stage 1, Stage 2 included injection of additional electron donors, buffers, supplemental acids, and supplemental bases but differed in that the KB-1 microbial culture was not added. Stage 2 took five months, after which there was a monitoring event and 3 months of incubation.

The groundwater chemistry and soil analyses showed that after bioaugmentation, the pH and redox conditions within the treatment area remained in a range that promotes biodegradation (pH in the range of 5.5 to 6.6, with a generally negative oxidation reduction potential (ORP)). In addition, decreased sulfate concentrations at all wells were consistent with active sulfate reduction in the treatment area, an indication of anaerobic conditions needed for reductive dehalogenation. Groundwater data indicated that reductive dechlorination occurred in the treatment area. Initial PCE concentrations at the upgradient edge (7640 μg/L; near the source) decreased by 95% after Stage 2 treatment. While contaminant concentrations in most wells remained low after treatment, concentrations in one well increased from a low of 180 μg/L after Stage 2 to 3540 μg/L. The cause was not clearly identified, but it suggests that treatment was not uniform throughout the treatment area. Ethene, the end product of dechlorination, increased in the treatment area from non-detectable to tens to hundreds of mg/L, and biomarkers (*Dhc* and *vcrA*) increased by a factor of 1000 from levels near or at the detection limit.

Soil data showed no reductive dechlorination within the treatment area prior to EK treatment. After Stage 2, PCE concentrations within the treatment area decreased by 78% to 99% (from baseline values ranging from >3300 μg/kg to >15,000 μg/kg), with significant improvements apparent by Stage 1. There were, however, no clear patterns of increased daughter products from the dehalogenation (TCE, 1,2-dichloroethene (DCE), VC). Post-Stage 2 samples showed quantifiable levels of *Dhc* and *vcrA*, which were not present at baseline or after Stage 1. The biomarkers appear at locations in the interior of the treatment area, which received an ample supply of electron donors during both stages.

#### 3.2.4. Lessons Learned

The data from the Jacksonville NAS site indicate that EK treatment is promising as a technology for diffusion-limited media. This is because electrokinetic flux is much less susceptible to limitations arising from subsurface heterogeneity and because the flux in low-conductivity materials is larger than can be achieved by hydraulic means. Consistent with the intended remedial mechanism, this study showed that reductive dechlorination was responsible for reductions in soil PCE concentrations in the LCZ (average: 88% (*n* = 9); range: 78% to 99%) over a duration of 22 months. Similarly, reductions in groundwater PCE concentrations averaged 81% (*n* = 6; range: 67% to 95%).

Subsequent work by Meinel et al. [65] examined the effects of EK bioaugmentation on the microbial communities at the Jacksonville pilot study using groundwater samples from the second stage. The authors concluded that the influence of EK bioremediation on the dechlorinating microbes is similar at both the laboratory and field scales and further supports the work by Cox et al. [64] regarding the ability of this technology to promote CVOC degradation.

However, technological advancements that facilitate the use of EK are necessary. For instance, the performance of the EK bioaugmentation technology depends on efficient migration of the amendments and microbial consortium through the subsurface. A better understanding of the mechanisms and factors affecting this migration is needed for optimal system design. At Jacksonville, the spatial distribution of lactate was estimated to be about twice as large as the zone reached by microbial amendments [65]. This is in line with previous EK bioaugmentation studies that showed that lactate transport was driven by electromigration and microbial transport by electroosmosis [90,91]. In general, electromigration is as much as an order of magnitude faster than electroosmosis [87]. The role of electrophoresis in the migration of bacteria has been suggested to be minor compared to electroosmosis [90].

However, even with a general understanding of which transport mechanisms are likely to govern migration of ionic amendments versus bacteria, a number of other factors can come into play, such as the type of bacteria and their capacity to sorb to the matrix [90], pore fluid chemistry, mineralogy, sorption of amendments to the clay, and physical properties of the clay (tortuosity and pore throat sizes) [87,91]. In addition, a thorough initial site characterization, as carried out by Adamson et al. [19], provided valuable information for planning and interpreting results from the EK study. A similar level of detailed post-treatment core sampling has not been reported for this study. Such a record would have provided important insights for evaluating the performance of this demonstration as well as a baseline for future EK studies.

Overall, the EK bioaugmentation treatment performance was comparable to other methods (e.g., enhanced bioremediation, thermal treatment) often applied to DNAPL source zones [92]. In the case of enhanced bioremediation based on hydraulic injection, EK bioaugmentation will likely be the better option for promoting amendment and microbe distribution in the LCZ. However, this benefit may be offset by other considerations, such as specialized knowledge needed for EK applications, pH control associated with EK electrodes, and other site-specific considerations.

Practical, on-site factors also come into play when making a final remedial decision. For example, upscaling the technology can present challenges. At Jacksonville NAS, the EK bioaugmentation treatment area was very small relative to the entire CVOC contamination. Deploying the appropriate density of electrode, injection, and monitoring wells for a much larger area may not be practical (M. Singletary, personal communication, 29 August 2022), although application at hotspots could be an advantageous use of this technology. Although EK bioaugmentation was not pursued for larger deployment at Jacksonville, the demonstration project provided proof of concept, valuable information on operational parameters, and data to support exploration of the mechanisms causing dispersal of the amendments.

### 3.3. Case Study: Naval Air Warfare Center (NAWC) Superfund Site

Located in West Trenton, NJ, the Naval Air Warfare Center (NAWC) is a former U.S. Navy facility that was used for jet engine testing from 1953 to 1998. Chlorinated solvent leakage and waste disposal practices resulted in CVOC contamination (primarily TCE) in fractures and the rock matrix at the site [66,68], in addition to jet fuel which also leaked into the subsurface. Groundwater contamination persists despite ongoing PAT operations. The presence of contaminants in the low-conductivity shale matrix is considered the major source of contaminants to the high-conductivity fractures over the long term.

Research on groundwater contamination and remediation at the facility has been ongoing since the 1980s. The U.S. Geological Survey (USGS) and several other federal, state, and private cooperating organizations (e.g., SERDP, United States Environmental Protection Agency (U.S. EPA), New Jersey Department of Environmental Protection, universities, consultants) have been studying the fate and transport of chlorinated solvents at NAWC [24,66–72]. The research includes the evaluation of natural attenuation due to biodegradation occurring at the site [93]. In particular, a bioaugmentation/stimulation field study was conducted from 2008 to 2010 to evaluate CVOC removal from the fractured rock and to evaluate the fate and transport of injected amendments [68].

#### 3.3.1. Site Geology and Hydrogeology

**Figure 5.**
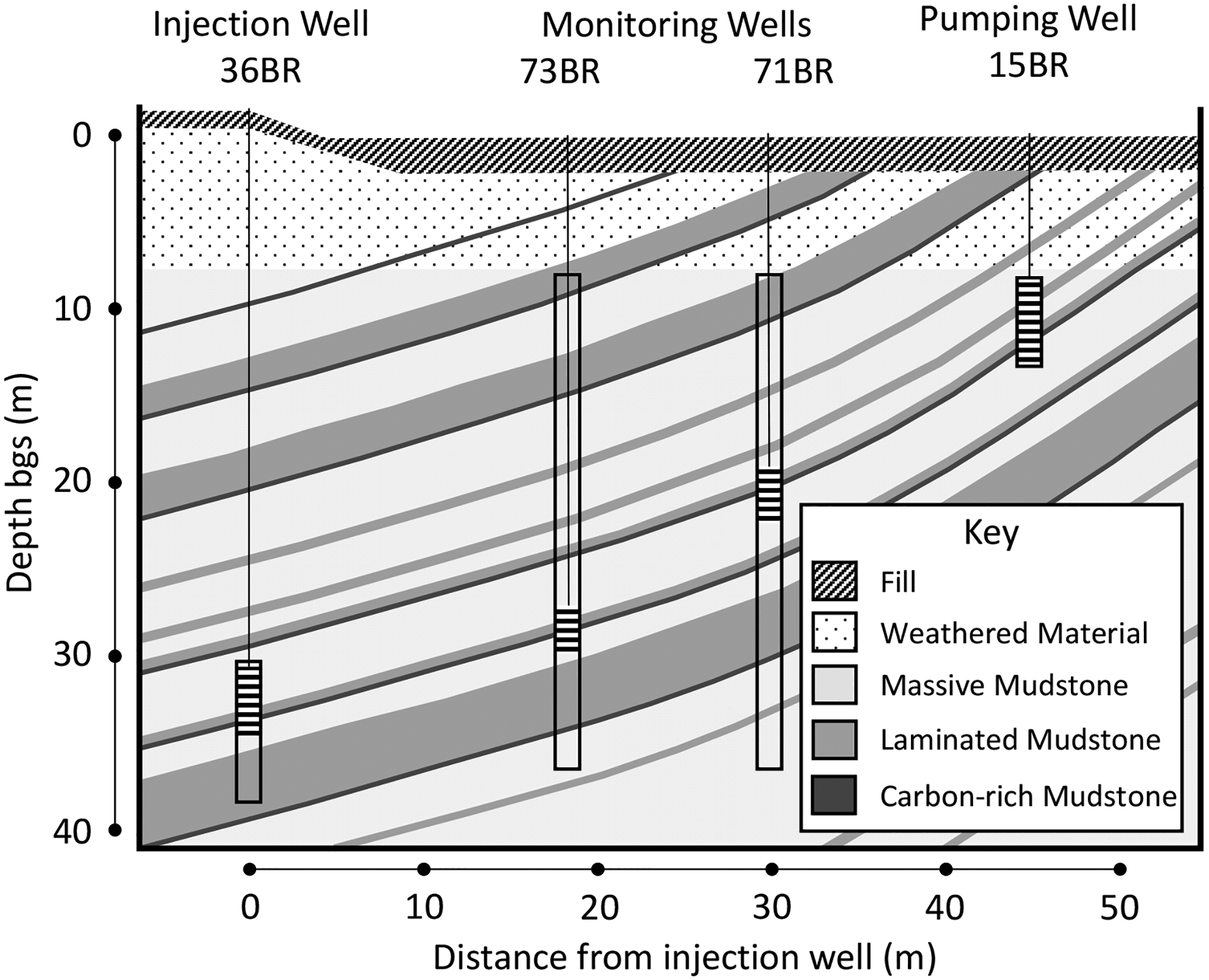
Cross section showing primary flow path from injection well 36BR to pumping well 15BR as indicated by the packer-isolated sampling intervals (horizontal line pattern). Adapted from Bradley et al. [96]. Geologic descriptions and primary flow path as noted by Révész et al. [68].

The NAWC site geology was described by Lacombe [94] and consists of gently dipping mudstones and sandstones (Figure 5). The upper 10 to 15 m are heavily weathered and fractured. They are underlain by unweathered mudstones [72,95] where groundwater flow occurs in fractures perpendicular to bedding. Groundwater has traveled up-dip for the past 17 years due to pumping [68]. In addition to the degree of lamination and bedding fractures, a key characteristic that varies among the mudstones is their high organic carbon content, which can be up to a few percent [70]. The conductivity of the unfractured mudstone at NAWC is low, as measured during field tests, and often below detection [67,72]. This indicates that the primary mechanism for contaminant transport in the rock matrix is diffusion [24].

#### 3.3.2. Nature of Contamination

Leakage and disposal of TCE over the course of decades resulted in subsurface contamination in both DNAPL and dissolved phases [66]. The contaminants migrated slowly downward into the unweathered bedrock. Two primary source areas are present at the site. In the more polluted source area, known as the West Area, TCE groundwater concentrations at or above 100,000 μg/L are detected in the mudstone at a depth of approximately 30 m [66,68,72]. Also present are TCE degradation products cDCE and VC. While the distribution of DNAPL is uncertain, it was identified in one core sample at a depth of 27 m [67], and high aqueous phase concentrations suggest its presence in other locations. Based on the analyses of rock cores from three boreholes in the West Area, it is estimated that 95% of the TCE is in the rock matrix [67,71], or ~70,000 kg [66].

The geology at the NAWC site results in a high degree of contaminant sorption to the rock matrix and thereby creates a substantial contaminant reservoir [24]. Core analyses have shown that TCE has only migrated a few centimeters into the rock matrix from the fractures [67]. As simulations suggest, the depth of penetration of TCE into the LCZ is far less than that expected without sorption; that is, more than 98% of TCE and more than 82% of cDCE mass in the matrix are located within 5 cm of the rock–fracture interface. Without sorption, TCE or cDCE would have diffused >30 cm into the matrix [24]. In addition, the mass of TCE stored in the matrix was estimated to be eight times greater with sorption than without [24]. The mass of TCE within the matrix and the limited distance that it has migrated away from the fractures are important information to consider when developing remedial designs at this or similar sites.

#### 3.3.3. Remedial History

The facility’s PAT system was installed in 1995 to remove the CVOCs from the groundwater and to hydraulically contain the contamination in the two main source areas [66]. It is hypothesized that the PAT system reversed the aqueous flow direction from down-dip to up-dip [97], but also increased downward movement of dissolved TCE because the wells were open in both shallow and deep units [67]. Over the first few years of operation, the PAT system was repeatedly expanded, reconfigured, and eventually consisted of eight wells. Since then, an estimate of the monthly pumped volume is ~7600 m^3^ [66,98,99].

Besides PAT, natural attenuation through biodegradation has been considered an important part of the overall site remediation. Natural attenuation is estimated to have removed about 500 kg/year of TCE beyond the 630 kg/year removed by the PAT system [100] over roughly the first decade of operation. Stable isotope data showed that TCE was enriched in ^13^C relative to the pure product, and this was considered indicative of natural biodegradation [68]. Nonetheless, Shapiro et al. [71] note that natural attenuation together with PAT would take hundreds of years to remove contamination to the point where the water would meet regulatory guidelines.

**Figure 6.**
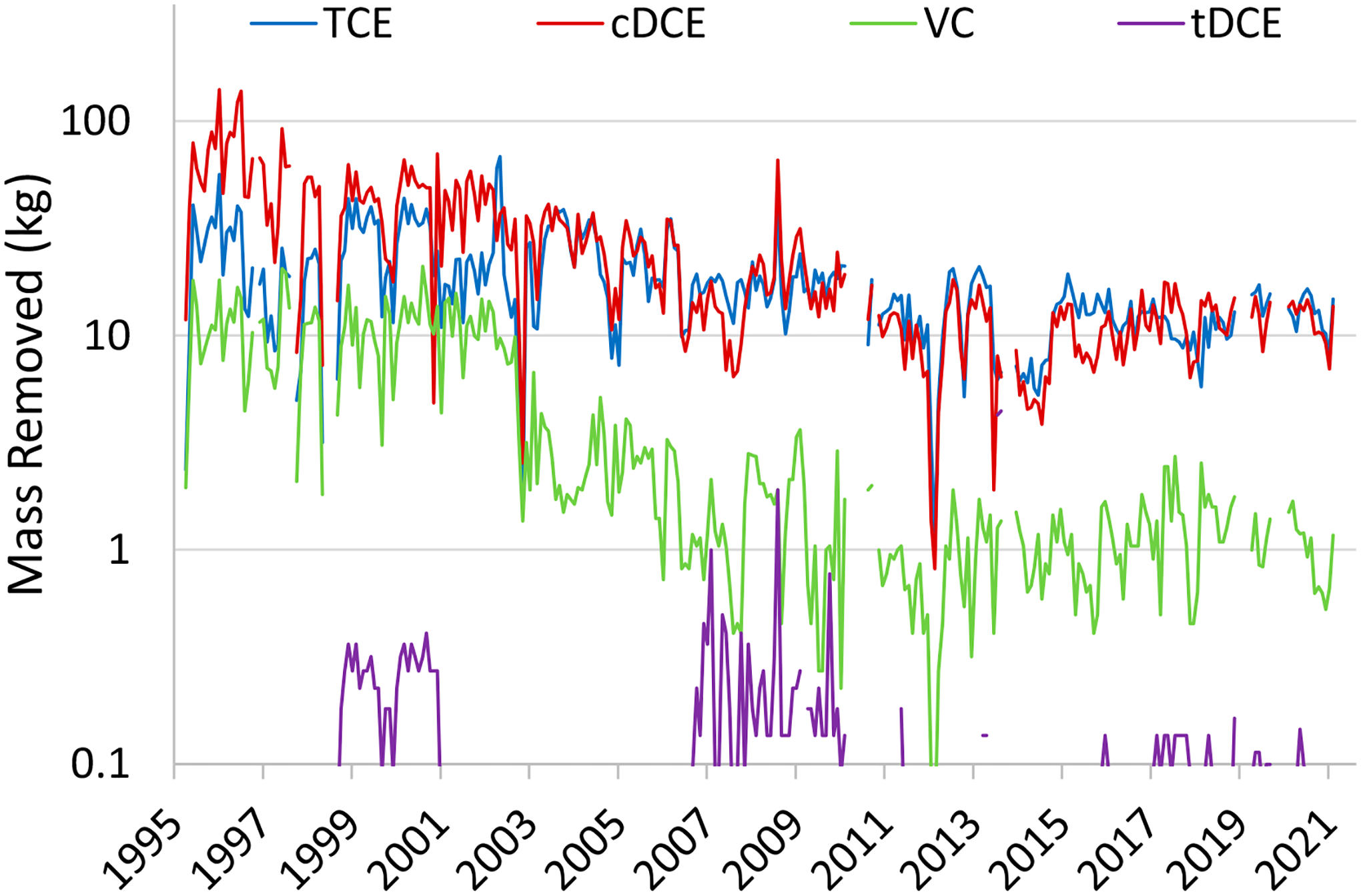
Monthly CVOC mass (kg) removed by the PAT system at the NAWC site. Data from 1995 to 2010 were taken from Lacombe [66], data from 2011 to 2020 were provided by the U.S. Navy, and data from 2020 to 2021 were taken from U.S. EPA monthly summary reports.

Lacombe [66] summarized the mass of CVOC removed by PAT at the site between 1996 and 2010. This data set was augmented with data ranging from 2011 to 2021 (Figure 6). During the first ~7 years of PAT operation (1995 to 2002), cDCE was the dominant CVOC in the combined treatment system influent, while later, cDCE was slightly below TCE (Figure 6). Overall, the rate of mass removal has been trending downward since the PAT system went into operation in 1995, as expected for a maturing system. However, since approximately 2015, the mass of recovered TCE and cDCE hovers around 10 kg per month, while the rate of VC removal is about one order of magnitude lower.

**Figure 7.**
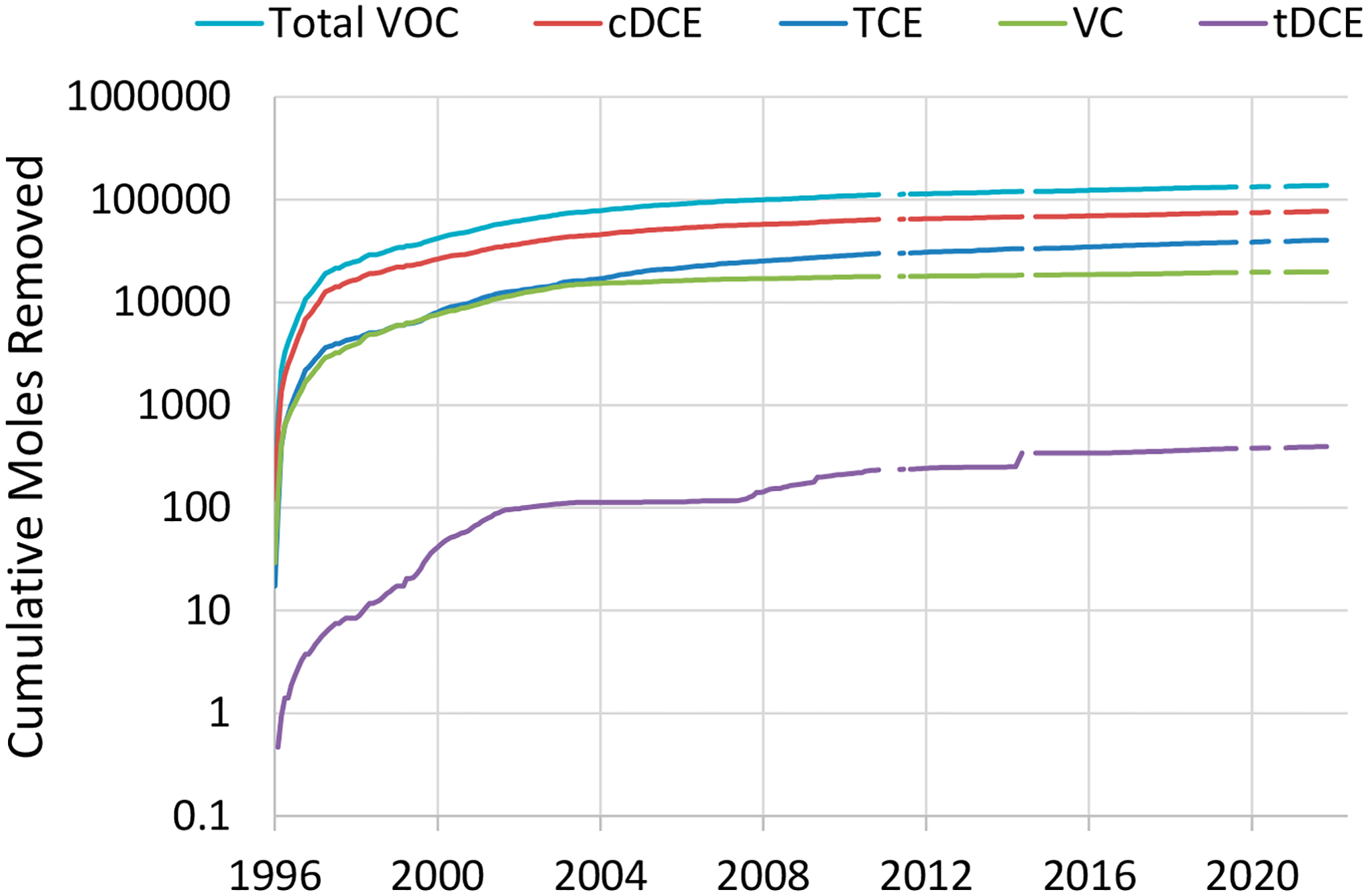
Cumulative moles of individual and total CVOC removed from NAWC by PAT from 1996 to 2021. Data from 1995 to 2010 were taken from Lacombe [66], data from 2011 to 2020 were provided by the U.S. Navy, and later data from 2020 to 2021 were taken from U.S. EPA’s monthly summary reports.

Figure 7 shows the cumulative moles of CVOCs extracted by PAT over the period of 1996 to 2021. The dominant species is cDCE over the entire observation time; however, the rate by which TCE is removed is greater (as signified by the steeper slope) than that of cDCE, particularly since 2004. The VC removal appears stagnant or is only slightly increasing since ~2003. The absence of appreciable levels of VC accumulating after 2003 suggests that biotic transformations of cDCE are not dominant at the site.

Results from Allen-King et al. [24], who analyzed boreholes for CVOC diffusion and determined degradation rates at NAWC, help to explain, at least in part, why cDCE is the dominant contaminant, as illustrated in Figure 7. First, abiotic degradation products of TCE were not observed (i.e., acetylene, which indicates abiotic TCE dechlorination, was not detected in any water samples). Second, nonchlorinated TCE biodegradation products (ethene or ethane) were also not detected in any of the samples associated with the study. Third, the results showed that microorganisms consistently degraded TCE and that its concentration was not limiting the biodegradation reaction rate. The TCE biodegradation rate was rapid (≥131 μg/L/d; following a zeroth order rate law), while cDCE biodegradation was slow, with a half-life of 5.8 years. The accumulation of cDCE resulted from rapid TCE biodegradation and slow cDCE biodegradation, which is not uncommon at CVOC impacted sites (e.g., [101]).

After a smaller-scale pilot test in 2005, another bioaugmentation test was conducted between 2008 and 2010 [68,71]. The bioaugmentation study aimed to increase the removal of TCE and its degradation products (including full dechlorination to ethene) and track the fate and transport of the amendments and reactants [68,97]. The electron donor solution injected into the treatment zone contained a mixture of emulsified soybean oil, sodium lactate, and a vitamin B-12 solution for growth stimulation. It also contained the KB-1 microbial consortium. Water enriched with deuterium (^2^H) acted as a conservative tracer. Although the augmenting agents were expected to act primarily in the HCZ fractures because of microbial pore size exclusion [70], the bioaugmentation indirectly targeted CVOCs in the LCZ by enhancing the degradation of contaminants already in the HCZ fractures. This created a stronger concentration gradient from the LCZ toward the HCZ and brought contaminants that diffuse back out from the matrix in contact with bioaugmentation amendments in the fractures.

Figure 5 shows the upgradient bioaugmentation injection well (36BR) that delivered the amendments, two intermediate monitoring wells (73BR and 71BR), and the downgradient pumping well (15BR). The TCE concentrations (in μg/L) in these four wells were 102,492 (36BR), 18,922 (73BR), 10,906 (71BR), and 4993 (15BR). A hydraulic connection with fluid flow along fracture zones was documented through aquifer testing [68], and the monitoring program was designed using results from groundwater flow modeling.

During the bioaugmentation test, increased δ^13^C values and decreased TCE concentrations were observed in the injection well and in monitoring well 73BR (~20 m from the injection well), where δ^13^C is an isotopic signature based on the ratio of ^13^C to ^12^C in the sample to that in a standard. Changes were seen seven months later in monitoring well 71BR (~30 m from the injection well) but not in the pumping well 15BR (~50 m from the injection well). DCE and VC showed initial depletion in ^13^C, indicating that they were formed as TCE degraded, followed by enrichment in ^13^C as they in turn were degraded. This pattern persisted for a month after injection, after which TCE concentrations remained too low for analysis at the injection well and eventually too low at monitoring well 73BR. The weighted average δ^13^C values over time, along with ethene concentrations, suggest that bioaugmentation led to reductive dechlorination that proceeded past VC to ethene at least at the injection well and monitoring well 71BR during the year after amendment injection [68].

Three weeks after bioaugmentation/biostimulation, the concentration of cDCE in well 36BR rose from less than 10,000 μg/L to approximately 150,000 μg/L, significantly higher than that of the TCE before injection [68]. Less pronounced spikes were observed in 73B, while cDCE concentrations in 71BR increased about a year later. The resulting rise in the total chlorinated ethene concentration is consistent with migration of TCE from the rock matrix into solution, followed by degradation to cDCE in the fracture water. As cDCE increased, TCE rapidly declined to undetectable before increasing again as cDCE concentrations began to drop and VC eventually began to increase.

A delayed peak in total CVOCs (TCE + cDCE + VC) was observed and was interpreted to be the result of the biodegradation of TCE that entered the system from the LCZ after the initial rapid biodegradation. The concentrations of CVOCs and the ^2^H tracer at 36BR plateaued for about a year, declined, and returned to pre-bioaugmentation levels approximately two years after injection. Daughter products VC and cDCE remained elevated in the first monitoring well (73BR) for about two years. At the second monitoring well (71BR), cDCE and VC remained elevated for two years while TCE was lower relative to prebioaugmentation, although δ^13^C for the CVOCs were at pre-bioaugmentation values [68]. For TCE specifically, data presented by Shapiro et al. [71] indicate that TCE concentrations in 36BR, 73BR, and 71BR were reduced for up to 5 years. However, there was no appreciable reduction at the pumping well (15BR), indicating the short distance over which the effects of the bioaugmentation were able to be discerned due to dilution at the pumping well along with consumption of amendments along the flowpath and over time. Variability in the microbial community may give rise to temporal and spatial variation in the rates and extent of biodegradation, including cDCE and VC. This in turn could affect the flux of CVOCs from the rock matrix [68].

Shapiro et al. [71] present a mass balance to estimate the transport of CVOCs from the rock matrix both before and after the bioaugmentation. The mass balance relies on the data from rock core analyses for initial mass of CVOCs in the rock matrix, results of the bioaugmentation experiment, and groundwater flow modeling. To estimate transport rates by back diffusion, Shapiro et al. [71] defined a mixing volume extending along the flowpath from 36BR to 15BR, representing the portion of the subsurface affected by the bioaugmentation. The rate of transport of CVOCs + ethene (normalized to TCE concentration) from the matrix into the fractures increased by approximately an order of magnitude after bioaugmentation, from 7 kg/year to 45 kg/year as one example of the results obtained. Additionally, transport rates per unit volume were shown to be spatially variable. At the injection well (36BR) transport rates increased from 2.18 kg TCE/m^3^-year before bioaugmentation to 13.3 kg TCE/m^3^-year after (based on 3 years of data). At well 73BR, partway towards the pumping well, the transport rates similarly increased from 1.25 kg TCE/m^3^-year to 10.2 kg TCE/m^3^-year. This one order of magnitude increase persisted over five years of monitoring after bioaugmentation started. Shapiro et al. [71] attribute the increased CVOC fluxes to back diffusion, desorption, and DNAPL dissolution. However, even though the increased transport rate in the post-bioaugmentation period is notable, it would still take decades to achieve TCE remedial goals [71].

#### 3.3.4. Lessons Learned

Work at the NAWC site shows the importance of site characterization in a fractured rock aquifer because groundwater flowpaths will be affected by the fracture network as well as pumping. There are also several other site-specific factors affecting contaminant transport—including migration into and out of LCZs, matrix organic carbon content, matrix porosity, and CVOC mass in the matrix. As shown by Allen-King et al. [24], understanding local mass distribution processes helps to understand site-wide treatment options, particularly when the various geologic, geophysical, hydraulic, and geochemical data are used in flow and transport models. Further, this site illustrates that a large fraction (~95%) of contaminant mass can reside in LCZs and may not be directly accessible to treatments that rely primarily on advection. Combined with the concentration–time series data collected over almost two decades of PAT operation, these data sets illustrate the impacts of back diffusion and highlight the implications for long-term stewardship if treatments that target the LCZs are not applied at the site.

The bioaugmentation pilot test was deemed successful at decreasing CVOC concentrations in the fractures. The resulting change in concentration gradient between the fractures and matrix promoted back diffusion; TCE was then degraded in the fractures after leaving the matrix. This experimental work illustrated how multiple lines of monitoring data (stable carbon isotopes, CVOC concentrations, a ^2^H tracer, microbial populations, and electron donor concentrations) can be used to evaluate both effectiveness and mechanisms of bioaugmentation.

In terms of performance at the NAWC site, the effects of the bioaugmentation diminished with distance from the injection well and over time. As the electron donor reservoir provided by amendment injection is depleted, the rate at which TCE is biodegraded in the fracture will decrease. Once all the electron donor is consumed, biodegradation rates will decrease and the TCE concentration in the fracture will increase due to back diffusion from the matrix. With increasing fracture TCE concentration, back diffusion will slow down because of the smaller concentration gradients between the fracture and the LCZ matrix. Therefore, repeat bioaugmentation treatments would be required, most likely over many years, to successfully remediate this site if bioaugmentation was implemented for site remediation [71].

### 3.4. Case Study: Brandywine DRMO Yard

The Defense Reutilization and Marketing Office (DRMO) Yard is in Brandywine, Maryland, approximately 80 km south of Baltimore. The DRMO site, ~3.2 hectares in size, is currently inactive but was used by the U.S. Navy and the U.S. Air Force (USAF) between 1943 and 1987 as a waste storage area. Environmental investigations began at the site in the mid-1980s and it was placed on the National Priorities List in 1999 [102]. It was also included as a case study in an ESTCP project that evaluated alternative endpoints at challenging sites [81].

Following more than a decade of soil and groundwater remedial activities, the persisting contaminants at this site have been identified in two distinct hydrogeologic horizons [74,102,103]: the vadose/smear zone at the top of the Brandywine aquifer, where 1,4-dichlorobenzene, 2-methylnaphtalene, naphthalene, iron, and manganese are present; and the aquitard at the base of the aquifer underlying the site, where PCE, TCE, and their degradation products cDCE and VC are back-diffusing into the aquifer above. The focus of this case study is back diffusion of the CVOC contamination, and the vadose zone contamination is omitted from further discussion. The final record of decision for the site [102] explicitly identifies back diffusion of contaminants in the Calvert Formation as a contaminant source to address in the list of remedial action objectives.

#### 3.4.1. Site Geology and Hydrogeology

The water-bearing Brandywine Formation overlies the Calvert Formation, an aquitard. The Brandywine Formation ranges from 6.4 to 9.1 m in thickness and is composed of heterogeneous layers of clay, silt, sand, and gravel. This formation is composed of four layers (from top to bottom): the Shallow Brandywine, a continuous clay with some silt, sand, and gravel 0.6 to 3.7 m thick; the Upper Intermediate Brandywine, a discontinuous fine sand with minor silt and clay 0 to 2.3 m thick; the Lower Intermediate Brandywine, a continuous water-bearing stratum of poorly graded sand with gravel 2.4 to 7 m thick; and the Deep Brandywine, a discontinuous oxidized and poorly graded sand and gravel less than 0.8 m thick. The depth to the top of the Calvert Formation ranges from 6.4 to 9.1 m and the boundary between it and the Brandywine Formation above is highly variable [73,102,104].

Groundwater at the site is typically less than 3.1 m deep and can be as shallow as 0.9 m. Prior to groundwater remediation activities that started in 2000, groundwater flow at the site was generally to the northwest [74,103]. The highest hydraulic conductivity in the Brandywine Formation is 0.065 cm/s and much lower in the underlying Calvert Formation aquitard (9.8 × 10^−7^ cm/s) [73].

#### 3.4.2. Nature of Contamination

**Figure 8.**
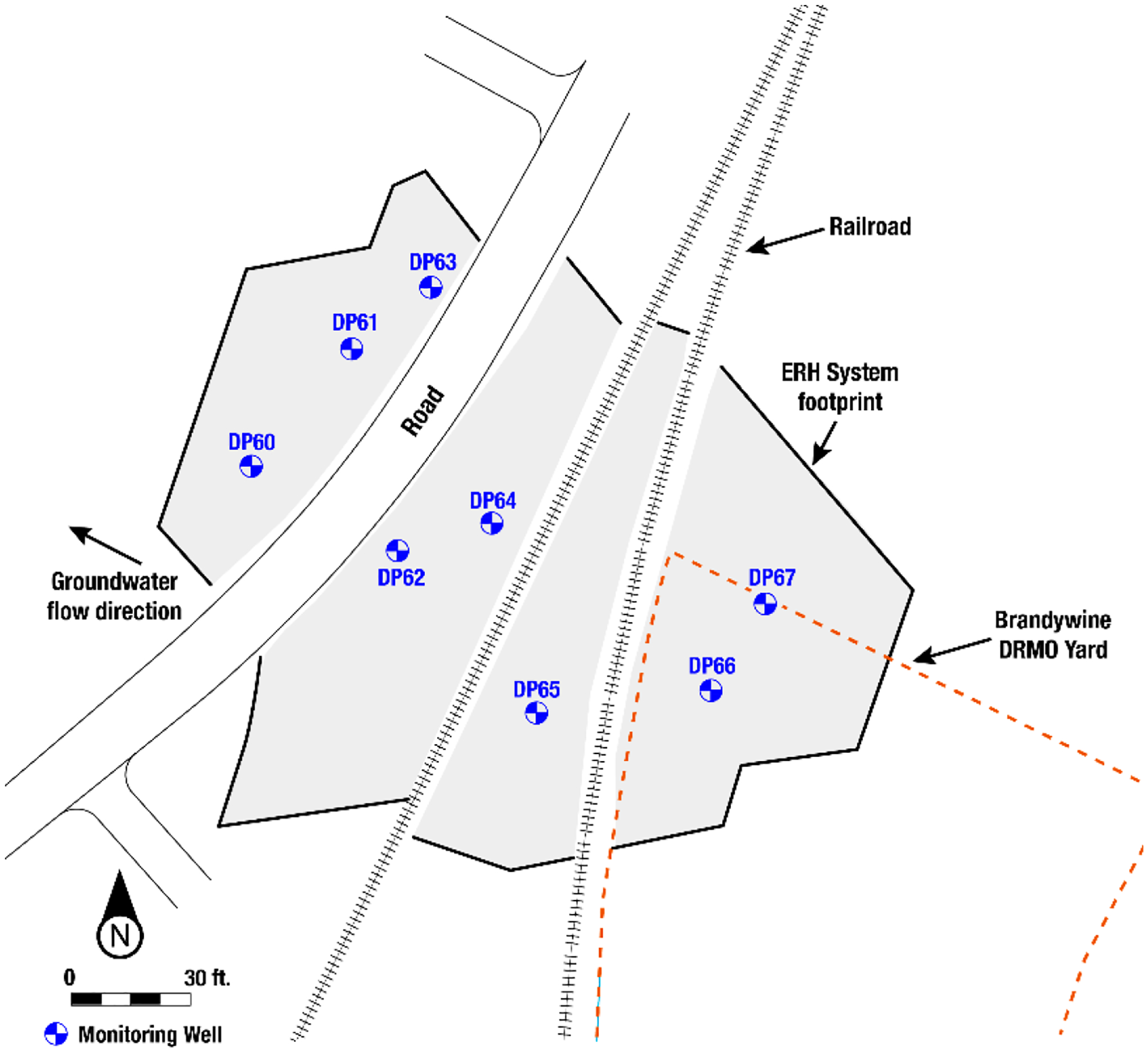
The Brandywine DRMO Yard with the ERH treatment footprint. Key surface features, groundwater flow direction, and location of monitoring wells are shown. All ERH vertical bored and sheet pile electrodes are located within the system footprint noted. Adapted from HGL [77].

Since the mid-1980s, multiple phases of site investigation and remediation have been conducted [105–107]. The contamination source was located near the northwest corner of the Brandywine DRMO yard (Figure 8). Investigations in 2010 did not confirm the presence of suspected DNAPL. More recent studies included identification and characterization of the role back diffusion plays in persistent CVOC contamination at the site.

Investigations in 2011 and 2012 [73] included 36 membrane interface probe (MIP) borings pushed into the clay of the Calvert Formation to map the distribution of CVOC in the subsurface. An electron capture detector (ECD) was used with MIP sampling to provide semi-quantitative, near real-time measures of contaminant concentration, and soil borings confirmed and further characterized subsurface contamination. Five flux wells were installed and screened to straddle the Brandywine–Calvert Formation boundary. Passive flux meters were used in these wells to measure contaminant flux at locations suggested by the MIP/ECD findings.

The MIP/ECD findings identified high concentrations of TCE, along with cDCE and VC, located primarily at the top of the Calvert Formation over an area of approximately 0.42 hectares, with the highest concentrations approximately 1 m into it. The highest mass flux of TCE at the site also corresponded to the Brandywine–Calvert boundary at a depth of approximately 6.7 m. Data collected during a rebound study [73] indicated that the primary mechanism of contaminant loading to the deep groundwater in the Brandywine Formation is back diffusion of TCE from the upper 2.4 to 3.1 m of the Calvert Formation [73,102].

#### 3.4.3. Remedial History

The first of two PAT systems consisted of a groundwater extraction trench and an extraction well in the northwestern corner of the site and removed approximately 230 kg of VOCs from 2000 to 2005 [103,108]. In 2006, in situ bioaugmentation and carbon substrate additions were selected to promote dechlorination with hydraulic gradient control [108]. The second PAT consisted of a groundwater extraction trench complemented by two permeable reactive barriers located downgradient from the first PAT. The reactive barriers were designed to promote enhanced in situ reductive dechlorination in the distal portions of the contaminant plume with three subsurface injections in 2008, 2010, and 2013–2014. The first injection was conducted to promote biotic degradation through bioaugmentation and biostimulation, but because of inadequate CVOC reduction, the second and third injections were conducted to promote abiotic degradation. Operation of the second PAT and the in situ bioremediation barriers reduced the size of the VOC plume from 7.81 hectares in 2007 to 0.49 hectares in 2017 [77]. The second PAT operated from 2008 to 2013 and extracted 47.3 million liters of water and removed 40.4 kg of VOCs [77]. Most contaminants in the distal plume met remediation criteria through these actions by early 2013 [104].

A subsequent site remediation goal was to reduce contaminant concentrations to below CVOC MCLs in the Calvert Formation and thereby eliminate back diffusion into the overlying Brandywine Formation [102]. Electrical resistance heating (ERH) was selected for this purpose and is an in situ remediation technology that is particularly effective at volatilizing and mobilizing CVOCs from LCZs such as the Calvert Formation. Heat introduced into the subsurface through ERH provides a means to volatilize and mobilize volatile contaminants, and then capture and remove them through vapor recovery and treatment [109]. The site’s ERH system consisted of two electrode designs: 43 pairs of vertical bored electrodes and 58 sheet pile electrodes. Sheet piles are less expensive to install and provide greater surface area for more efficient heating but could not be installed (driven) through the rail track beds given subsurface composition and because of limited overhead clearance near power lines (W. Burris, personal communication, 31 May 2022).

All vertical bored electrodes and sheet pile electrodes were installed to a depth of approximately 11.9 m bgs, approximately 2.4 to 3.1 m into the Calvert Formation. The effective thermal treatment interval was continuous from approximately the top of the aquifer to at least a meter into the underlying aquitard. A total of eight groundwater monitoring wells (Figure 8) and 17 temperature monitoring points provided data to evaluate subsurface treatment effectiveness. Above-ground vapor treatment was also monitored for treatment efficacy [77]. The ERH system operated for six months, from April 2019 to October 2019. The treatment endpoint was defined as reaching the TCE MCL in a single groundwater sample collected from the monitoring wells during thermal treatment. If some monitoring wells were to meet the criteria before others, electrodes near the affected groundwater monitoring well would be shut down [79].

The ERH system at the Brandywine DRMO Yard was shut down in October 2019 when multiple lines of evidence indicated site cleanup criteria had been achieved. The average subsurface temperature at the site from June 2019 to October 2019 was 106.3 °C. Mass removal rates declined in mid-August, indicating that the contaminant mass had significantly decreased, and that ERH had reached diminishing returns. Based on photoionization detector analysis of vapors recovered during ERH operations, an estimated 798 kg of VOCs were removed during remediation [76].

**Figure 9.**
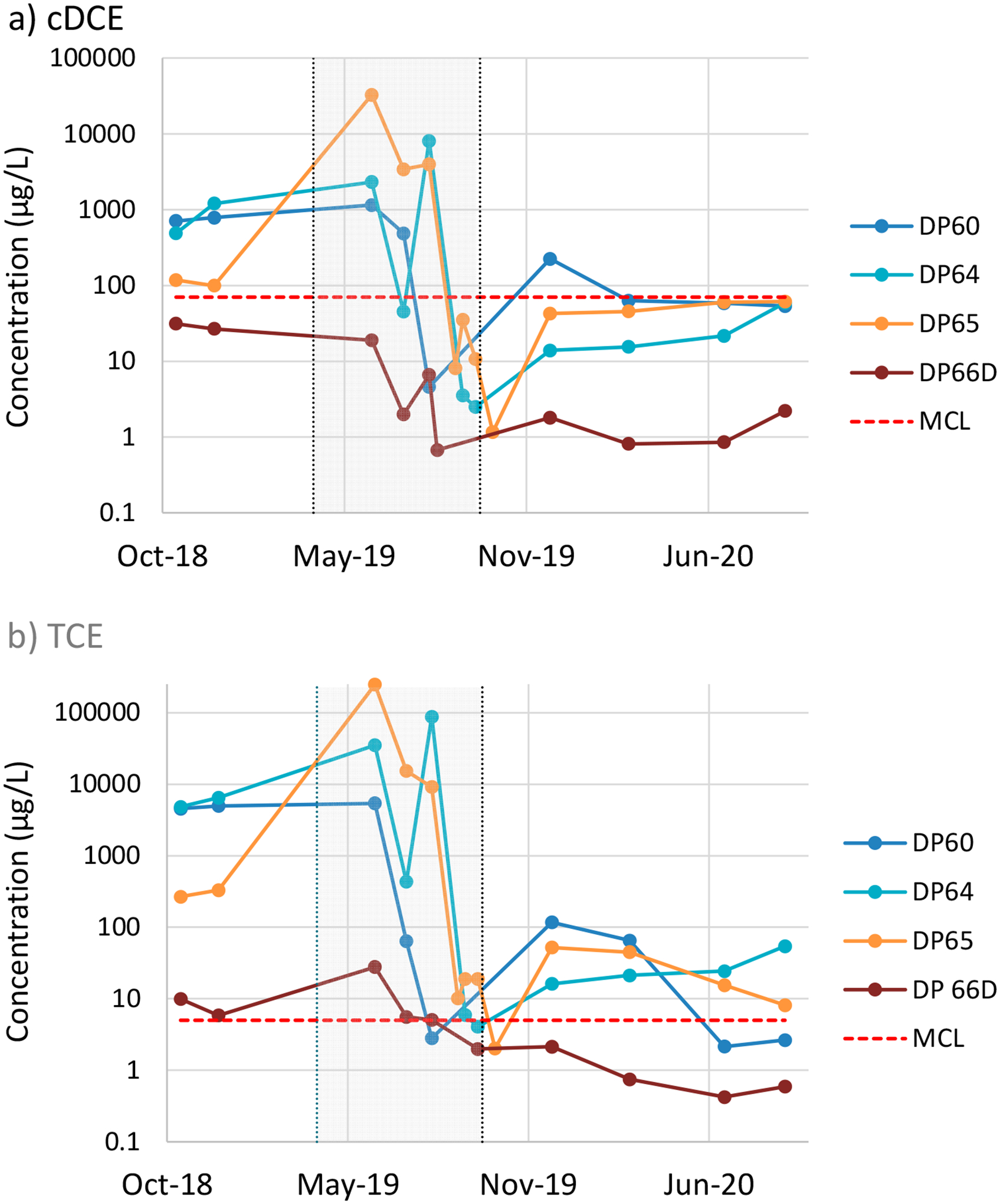
Concentrations of (**a**) cDCE and (**b**) TCE in selected groundwater monitoring wells located in the ERH Treatment Zone at the Brandywine DRMO Yard. The ERH thermal treatment system operated from April to October 2019 (gray area). The red dotted line indicates the MCL of the respective compound. Data from HGL [76,78].

Groundwater contaminant sampling results [76,78] from November 2018 (pre-treatment) through to September 2020 (post-treatment) are illustrated in Figure 9a for cDCE and Figure 9b for TCE. The post-ERH well sampling results for selected wells show that cDCE concentrations remained near or below their respective MCLs in most wells. For TCE, only wells DP60, DP66S (data not shown), and DP66D remained below the MCL. The baseline TCE concentration in well DP60 was much higher (~5000 μg/L) than that in DP66S and DP66D (~300 and ~10 μg/L, respectively), indicating that ERH treatment was particularly effective at DP60. For cDCE, an upward trend in concentration after treatment is evident in all wells except DP60, although only one well (DP67) has thus far exceeded the cDCE MCL. In the case of TCE, an upward trend in concentration is evident in only two wells (DP62 and DP64), while decreasing or relative stable trends are evident in the remaining wells. However, only three wells (DP60, DP66S, and DP66D) remained below the TCE MCL over the last two sampling events. Overall, Figure 9 shows significant declines in contaminant concentrations due to the remediation activities. All wells but one (DP61) met MCLs by the end of thermal treatment and shortly thereafter. However, contaminant concentrations rebounded above MCLs in some locations by late 2020, about one year after ERH treatment. The reason for the rebound is uncertain and may include a potential influx of contaminants from upgradient or incomplete treatment of contaminants in the LCZ.

#### 3.4.4. Lessons Learned

Treatment by ERH was successful in meeting the remedial criteria during and immediately after the treatment. Groundwater data showed that MCLs were reached in the Calvert Formation (the LCZ) at almost every location. Thermal treatment by ERH on average reduced concentrations by more than two orders of magnitude, which is consistent with previous thermal treatment evaluations (e.g., [92]). However, longer-term monitoring indicates contaminant rebound. Although the amount of contaminant mass removed by ERH will likely reduce the duration of site restoration, the post-ERH operation groundwater monitoring suggests continued back diffusion in most parts of the treatment zone.

Arguably, certain treatment endpoint criteria were set fairly low, i.e., an area was considered sufficiently treated when a sample from one well in the area indicated contaminant concentrations below MCLs [79]. It is likely that a longer ERH treatment duration would have further depressed the contaminant concentration in the treatment zone. Additionally, it is unclear if the observed rebound might be, at least in part, related to influx of contaminated groundwater from upgradient, perhaps in areas of the source zone not sufficiently treated by ERH. Additional investigation and monitoring would be necessary to further evaluate these issues and to explore if this site might at this point be more amendable for treatment by less intensive remedial approaches, such as managed natural attenuation.

## 4. Discussion

It may be appropriate to assume back diffusion is a relevant factor with a site hydrogeologic conceptual model that identifies HCZs and LCZs, coupled with tailing in concentration–time series data from either monitoring or pumping wells. However, other mechanisms besides back diffusion can contribute to contaminant rebound and sustain contaminant concentrations in groundwater, including slow advection, desorption, and DNAPL dissolution. Moreover, it is likely that more than one of these mechanisms may be a significant factor in plume persistence. This is particularly true at DNAPL sites where it is likely the case that both DNAPL dissolution and back diffusion are relevant and concurrent features over some finite duration as the site ages. As noted by Schaefer et al. [58], without sufficient data it can be difficult to determine which mechanisms are responsible for plume persistence. Schaefer et al. [58] also noted that contaminant migration from upgradient contaminant sources after treatment too often remains a source of uncertainty that impedes assessment of conditions in the treated area, and the case studies at Jacksonville NAS and Brandywine serve as examples of that herein. Because of these challenges, there are limited well-documented field sites where treatment of back diffusion can be studied in detail.

The four case studies evaluated herein were in large part selected because of the extensive efforts invested to assess the spatial distribution of contaminant mass relative to the hydraulic conductivity distribution. Diffusion is a slow process and contaminant distributions due to diffusive flux occur over relatively small distances; consequently, high-resolution vertical samples are important for back diffusion assessment. In particular, high-resolution soil cores are valuable for defining contaminant profiles as illustrated at the PFC [18], Jacksonville NAS [19], and NAWC [67] sites. Vertical spacing between samples needs to be of the order of decimeters to properly assess direction and magnitude of diffusive flux. This can be an expensive characterization task to complete, which may explain why high-resolution soil cores are more often collected during research activities, but less often in practice. Other types of high-resolution samples are also valuable, such as MIP as demonstrated at the Brandywine site [104]. Moreover, our review highlights a deficit with respect to high-resolution sampling, soil core or otherwise, after remediation. Such information is needed to help evaluate changes in contaminant spatial distributions and diffusive flux after treatment, particularly for treatments such as EK and ERH that are less susceptible to limitations in LCZ transport. The Brandywine site serves to illustrate the point. Samples from high-resolution soil cores could provide useful information for evaluating the performance of remedial measures taken to address back diffusion. Relying solely on groundwater concentrations provides information about concentration in the HCZs, but insufficient information about contamination in LCZs and back diffusion processes, which may control contaminant rebound and persistence in groundwater. This same feature may be a factor at the PFC site as well. More data are needed to evaluate the longevity of concentration reductions in the HCZs prior to concentration rebound driven by back diffusion from the LCZ layers.

Biostimulation and bioaugmentation were used at the PFC, Jacksonville NAS, and NAWC sites. Advective injection of amendments into the HCZs was used in the case of the first and last, while EK was used in the case of Jacksonville NAS. Results from the NAWC site indicate that reductions in HCZ concentrations promote mass transfer from the LCZ, but that repeated treatments would be needed over many years to effectively remediate the site [68,71]. A similar response can be expected at the PFC site, given the conceptual similarity in the underlying amendment and contaminant transport mechanism (i.e., increased back diffusion of contaminants from the LCZ due to increased concentration gradients from amendment driven reductions in the HCZ concentration), but presumably the longevity may be less at the PFC site given the nature of the LCZ architecture. The EK-based delivery mechanism used at Jacksonville NAS is not subject to limitations arising from the need for amendment advective transport. Results from the pilot test indicate that EK was successful in amendment delivery to promote biodegradation in the LCZ, with substantial decreases in PCE concentration [64]. However, site-responsible parties have suggested that advective injection of amendments will likely be used more broadly across the site, presumably because the cost of EK treatment outweighs the benefit when applied on a larger scale.

Several citations have suggested or demonstrated that degradation in the LCZ can have a significant effect on reducing plume persistence due to back diffusion [110–112]. Shapiro et al. [70] ruled out enhanced biotic degradation as a significant factor in the rock matrix at the NAWC site because the range in rock pore throat diameters was not sufficiently large to accommodate the size range for the microbes, and because substrate transport into the matrix would be limited by forward diffusion. As demonstrated by Shapiro et al. [71], degradation in the HCZ substantially increases mass transfer from the LCZ. However, in this scenario, site restoration is still limited by the slow rate of diffusional transport, but this slow rate means that even minor rates of LCZ degradation can be significant over long durations. Cox et al. [64] did not address pore size exclusion in their summary of the EK demonstration at the Jacksonville NAS, nor was it apparently evaluated at the PFC site. Previous work has suggested that pore size exclusion was not a limiting factor based on pore size analysis at three sites with clayey till [113]. Moreover, Lu et al. [113] suggest that microbes can adapt to space limitations. At the Jacksonville NAS site, biomarkers which could be used as direct evidence of microbes in LCZ pores were not detected in soil samples collected prior to the demonstration, nor were they detected after Stage 1. They were, however, found in five of nine core locations after Stage 2 within the interior of the test cell where better amendment coverage was obtained.

As highlighted in the EK pilot study at Jacksonville NAS and ERH treatment at Brandywine, complete contaminant mass removal from secondary sources responsible for back diffusion may not always be possible, even in the case of treatment technologies that are less susceptible to limitations arising from low hydraulic conductivity. While the reasons for this may vary depending on the site-specific conditions under which the remedial technology was applied, the result is nonetheless the need for another remedial treatment application with the same or different technology, or a different remedial management strategy altogether. While the potential need for a phased approach to remediation at complex sites is not new (e.g., [114]), it does highlight a question that may be asked if complete contaminant removal is not considered feasible: what benefit is achieved by partial contaminant mass removal from LCZs? This is a familiar question in the context of primary DNAPL source zone remediation, and a similarity can therefore be drawn between primary and secondary source zones in this respect. A summary of potential benefits associated with partial mass removal from DNAPL source zones was provided by the U.S. EPA [115]. These are applicable to partial mass removal from secondary source zones too, and include reduced longevity, reduced mobility/contaminant mass flux, enhanced efficiency and effectiveness of complementary technologies, economic benefits, and environmental stewardship. At the least, evaluating the first three of these depends on the spatial contaminant distribution in the LCZ after treatment, and again highlights the importance of post-treatment high-resolution sampling data.

## 5. Conclusions

A review of the available literature for case studies on remediation of plume persistence due to back diffusion was conducted, and four sites were highlighted: the PFC and Jacksonville NAS sites in Florida, the NAWC in New Jersey, and the Brandywine DRMO Yard in Maryland. For the first three case studies, information was integrated across multiple publications in the peer-reviewed literature to focus on the topic of back diffusion remediation. Moreover, recent monitoring data were summarized for the PFC and NAWC sites, which adds to the previously published results. Three general conclusions are made based on this review. First, it is difficult to assess the significance of back diffusion without sufficient data to distinguish between multiple factors contributing to contaminant rebound and plume persistence. Second, high-resolution vertical samples are decidedly valuable for back diffusion assessment. Third, complete contaminant mass removal from back diffusion sources may not always be possible. Partial contaminant mass removal may nonetheless have potential benefits, similar to partial mass removal from primary DNAPL source zones.

The case studies summarize remedial strategies used at sites where back diffusion of CVOCs is occurring. They highlight the outcomes and lessons learned for a range of remediation approaches and geological settings, as well as important limitations. As current and new remediation technologies are implemented, the lessons noted here improve the likelihood that site managers can remediate sites when back diffusion hampers cleanup efforts. Altogether, these advances will contribute to sustainable water supplies and healthier ecosystems.

Finally, our review highlights that a relatively small number of sites have been studied in sufficient detail to fully evaluate remediation of back diffusion. Relying too heavily on just a few sites hampers the assessment of back diffusion as a nationwide issue. Further, assessing the extent to which more innovative remedial strategies that target LCZs have been successful at reducing back diffusion is challenging without sufficient data for a wide variety of geographically diverse sites. Our review also highlights that assessment of LCZ spatial contaminant distributions after remediation treatment is lacking. Such information is needed to fully evaluate benefits of partial contaminant mass removal from LCZs. Consequently, we recommend the development of more case studies, with a focus on changes in LCZ contaminant distributions in sufficient detail after remediation to evaluate the effects on back diffusion flux and longevity.

## Figures and Tables

**Table 1.** Summary of sites exhibiting back diffusion.

Site Name and Location	Primary COC ^1^	Geology	Remediation	Key Points	References
Tucson International Airport Area, Tucson, AZ	Trichloroethylene (TCE), 1,1-dichloroethylene (DCE), chloroform, and chromium	Alluvial sediments interbedded locally with volcanic units (flows, tuffs, etc.)	Pump and treat (PAT), soil vapor extraction, hydraulic containment, in situ chemical oxidation (ISCO)	Rate-limited DNAPL dissolution, rate-limited sorption, and back diffusion contribute to persistent subsurface contamination at this site. Although the effect of back diffusion alone has been modeled at the site, the inability to isolate DNAPL as a source limits back diffusion assessment.	[13,43–45]
Lawrence Livermore National Laboratory, Livermore, CA	TCE, tetrachloroethylene (PCE)	Primary alluvial clay, silt, sand, and gravel	PAT, soil vapor extraction, bioremediation pilot test	Early modeling work indicated that diffusion and LCZ architecture are important factors controlling PAT duration; applied PAT optimization. Volatile organic compound (VOC) concentrations and plume extent appear to be stabilizing or declining, though back diffusion into the HCZ is still a concern. Eight-year bioremediation test in a fractured, cemented conglomerate with limited recharge; persistence of ethene taken as evidence that degradation rate was comparable to diffusive flux.	[46–50]
Dover Air Force Base (AFB), Dover, DE	TCE, PCE, cis-1,2-dichloroethylene (cDCE), 1,1,1-trichloroethane, vinyl chloride (VC)	Sand and silt overlying an ~5 m thick silt and silty clay loam aquitard	Excavation of DNAPL-contaminated surface soils, pilot tests of PAT in isolated test cells	Core samples taken in aquifer and aquitard four times, including pre-PAT, immediately post-PAT, and in subsequent years. Contaminant gradients in core samples from aquifer suggest back diffusion as a major mechanism responsible for rebound and tailing.	[11,51,52]
Edwards AFB, CA	PCE	Fractured granitic bedrock	Bioaugmentation with groundwater recirculation	In shallow fracture zone, complete DNAPL removal and no rebound. Lack of rebound after DNAPL removal suggests that DNAPL dissolution was the primary means of contaminant persistence. In deep fracture zone, less DNAPL removal and rebound were observed. Back diffusion, while acknowledged to be possible, was not explicitly targeted for research.	[53,54]
Watervliet Arsenal, Watervliet, NY	PCE and cDCE, with lesser TCE and VC	Fractured shale bedrock	ISCO using potassium and sodium permanganate	Average PCE reductions in a pilot test were >96% and stable isotope data indicated VOC destruction, but concentration rebounded to pre-treatment levels after the end of the test. Did not meet targets for permanganate distribution and residence time in a full-scale implementation, and experienced persistent clogging. Careful site characterization, including rock oxidant demand (which may affect diffusive flux due to mineral precipitation), can help with determining feasibility and planning remedial design.	[55,56]
Connecticut site (undisclosed location)	TCE	Sand aquifer; clayey silt aquitard	Steel sheet pile enclosure to isolate DNAPL	Core samples were taken from aquifer and underlying aquitard. DNAPL was isolated from the aquifer. TCE contamination was modeled, and contamination is expected to persist above MCL for centuries. Additional information on site and current concentrations/remedial activities was difficult to obtain due to undisclosed site name and location.	[14,57]
Calf Pasture Point, Naval Construction Battalion Center, North Kingston, RI	TCE	Sand, silt, and till over fractured bedrock	Monitoring only	Evaluation of a field test for back diffusion assessment. Field test and modeling simulations indicate rebound due to back diffusion. Laboratory tests with rock material showed abiotic dechlorination of TCE to cDCE.	[22,58]

Note(s):

1 COC: contaminants of concern.

**Table 2.** Summary of case studies.

Site Name and Location	Primary COC	Geology	Remediation	Key Points	Key References
Precision Fabricating and Cleaning (PFC), Cocoa, FL	TCE	Sand and silt with clay lenses	PAT and enhanced bioremediation	Pollution persisted due to back diffusion from LCZ clay layer. Two rounds of enhanced bioremediation reduced effects of back diffusion and possible DNAPL in the source zone.	[57,59–63]
Jacksonville Naval Air Station (NAS), Jacksonville, FL	PCE, cDCE, and VC	Layers of sand and clay	Electrokinetic (EK)-enhanced bioaugmentation	High-resolution core samples showed forward and back diffusion profiles. LCZ treatment pilot test used EK to deliver amendments for biodegradation. PCE decreased in treatment area.	[19,64,65]
Naval Air Warfare Center (NAWC), West Trenton, NJ	TCE	Fractured mudstones and sandstones with high organic carbon content	PAT, bioaugmentation delivered to HCZ	Bioaugmentation degraded TCE in HCZ fractures. High organic carbon content of LCZ retarded contaminant forward and back diffusion. Increased transport of contaminants out of the LCZ as a result of steeper back diffusion gradient. Repeated bioaugmentation would be needed over many years.	[24,66–72]
Brandywine Defense Reutilization and Marketing Office (DRMO) Yard, Brandywine, MD	Aquitard—TCE, cDCE, VC, PCE	Layers of clay, silt, sand, and gravel	Electrical resistance heating (ERH) thermal treatment	Site cleanup criteria and remedial action objectives were achieved. Mass removal rates diminished after 4.5 months. Rebound observed after shutdown—unclear if rebound was caused by inflow from upgradient.	[73–79]

## Data Availability

No new data were created in this study. The data is available in the references noted.

## Abbreviations

^2^H: Deuterium
^13^C: Carbon isotope with an atomic mass of 13 amu
AFB: Air force base
AS/SVE: Air sparging and soil vapor extraction
bgs: Below ground surface
cDCE: Cis-1,2-dichloroethene
COC: Contaminant of concern
CSIA: Compound-specific isotope analysis
CVOC: Chlorinated volatile organic compound
DCE: 1,1-dichloroethylene
*Dhb*: Dehalobacter
*Dhc*: Dehalococcoides
DNAPL: Dense non-aqueous phase liquid
DRMO: Defense Reutilization and Marketing Office
ECD: Electron capture detector
EK: Electrokinetic
ERH: Electrical resistance heating
ESTCP: Environmental Security Technology Certificate Program
HCZ: High-conductivity zone
ISCO: In situ chemical oxidation
LCZ: Low-conductivity zone
MCL: Maximum contaminant level
MIP: Membrane interface probe
NAPL: Nonaqueous phase liquid
NAS: Naval air station
NAWC: Naval Air Warfare Center
ORP: Oxidation reduction potential
PAT: Pump-and-treat
PCE: Tetrachloroethylene
PFC: Precision Fabricating and Cleaning
SERDP: Strategic Environmental Research and Development Program
TCE: Trichloroethylene
TOC: Total organic carbon
USAF: United States Air Force
U.S. EPA/EPA: United States Environmental Protection Agency
USGS: United States Geological Survey
VC: Vinyl chloride
*vcrA*: Vinyl chloride reductase
VFA: Volatile fatty acid
VOC: Volatile organic compound
δ^13^C: Isotopic signature based on the ratio of ^13^C to ^12^C in the sample to that in a standard
